# Lighting up Macrophage Reprogramming Assists Immunosuppressive Niche Modulation in Primary Tumors and Tumor‐Draining Lymph Nodes of Breast Cancer

**DOI:** 10.1002/advs.75593

**Published:** 2026-05-06

**Authors:** Yingbo Li, Di Chang, Jie Yang, Ying Bai, Min Chen, Daoshuang Li, Zuoyu Xu, Jiaxin Yuan, Xiaoxuan Xu, Zhiqi Zhang, Yize Li, Zixin Chen, Jinbing Xie, Zebin Xiao, Shenghong Ju

**Affiliations:** ^1^ Department of Radiology Zhongda Hospital Medical School of Southeast University Nurturing Center of Jiangsu Province for State Laboratory of AI Imaging & Interventional Radiology Nanjing China; ^2^ Department of Radiology Zhongda Hospital Medical School of Southeast University Nurturing Center of Jiangsu Province for State Laboratory of AI Imaging & Interventional Radiology Collaborative Innovation Center of Radiation Medicine of Jiangsu Higher Education Institutions Nanjing China; ^3^ Department of Pharmacology Jiangsu Provincial Key Laboratory of Critical Care Medicine School of Medicine Southeast University Nanjing China; ^4^ Department of Radiology The First Affiliated Hospital of Kangda College of Nanjing Medical University/The First People's Hospital of Lianyungang Lianyungang Jiangsu China; ^5^ Department of Ultrasound The Fourth Hospital of Harbin Medical University Harbin China; ^6^ Department of Nuclear Medicine The Fourth Hospital of Harbin Medical University Harbin China

**Keywords:** breast cancer, immunotherapy, macrophage polarization, NIR‐II, patient‐derived fragments, phagocytose, tumor‐draining lymph nodes

## Abstract

The recurrence and metastasis of breast cancer are driven by immunosuppressive myeloid cells in tumors and lymph nodes. The inherent heterogeneity of myeloid cells poses a significant challenge for real‐time imaging and precise treatment. In this study, we developed microenvironment‐responsive second near‐infrared (NIR‐II) biomimetic nanoparticles (PA NPs) to spatiotemporally modulate macrophage‐mediated immunotherapies. These NIR‐II activatable “off–on” PA NPs accumulate in tumors and lymph nodes, enabling controlled immune activation. By incorporating colony‐stimulating factor 1 receptor inhibitors, PA NPs repolarized M2‐like macrophages toward the pro‐inflammatory M1‐like phenotype. The subsequent production of nitric oxide (NO) specifically illuminated the NIR‐II fluorescence of PA NPs, thereby providing dynamic visualization of macrophage migration and polarization in vivo. Moreover, PA NPs functionalized with anti‐CD47 antibodies selectively bound to tumor cells, blocked the CD47‐SIRPα “don't eat me” signal, and actively reprogrammed macrophages to enhance phagocytic clearance of tumor cells. In multiple breast cancer models, these nanoparticles effectively remodeled immunosuppressive niches and induced durable anti‐tumor immunity, which was further validated using patient‐derived tumor and lymph node fragments. Collectively, this strategy integrates NIR‑II–guided diagnosis and macrophage reprogramming therapy to remodel the immunosuppressive microenvironment across primary and metastatic niches, offering a potent immunotherapeutic approach against breast cancer.

## Graphical abstract

This study develops an intelligent NIR‐II‐responsive nanoplatform to combat immunosuppression in breast cancer. It enables real‐time visualization of macrophage activity via “off‐on” fluorescence while simultaneously reprogramming macrophages through the CSF‐1R and CD47‐SIRPα pathways. This integrated strategy effectively remodels the tumor and lymph node microenvironment, inducing potent and durable anti‐tumor immunity in preclinical models and patient‐derived tumor and lymph node fragments.

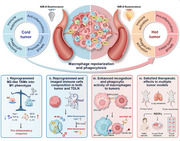

## 1 Introduction

Triple‐negative breast cancer (TNBC) is the most aggressive subtype of breast cancer and is notorious for its rapid progression, early systemic spread, and lack of effective targeted therapies [1, 2, 3, 4]. Its poor prognosis stems from a profoundly immunosuppressive tumor microenvironment (TME) that blunts the efficacy of chemotherapy, targeted therapy, and emerging immunotherapies [5, 6]. Despite intense efforts, clinical strategies to remodel the TME have yielded only modest benefits, partly because they concentrate almost exclusively on primary tumors while neglecting tumor‐draining lymph nodes (TDLNs) [7, 8, 9, 10]. TDLNs are not passive conduits of metastasis but rather decisive regulators of systemic immunity and early metastatic seeding [11, 12, 13, 14]. During tumor progression, breast cancer cells secrete cytokines and growth factors that induce lymphangiogenesis and recruit immunosuppressive immune subsets to the TDLNs, establishing a premetastatic niche [15, 16]. Once colonized, the tumor cells reinforce immunosuppression and accelerate their dissemination to distant organs [17]. These insights reveal that sustainable therapeutic benefits require disruption of the cooperative immunosuppressive axis linking the TME and TDLNs.

Tumor‐associated macrophages (TAMs) dominate both tumors and TDLNs [18, 19, 20]. In TNBC, the abundant infiltration of M2‐like TAMs drives immune evasion, suppresses cytotoxic T‐cell responses, and tightly correlates with recurrence, metastasis, and poor patient survival [21, 22]. The CSF‐1/CSF‐1R axis is a key regulator of TAM survival and polarization [23, 24, 25]. Inhibitors such as PLX3397 have shown preclinical potential by depleting or reprogramming M2‐like TAMs [26, 27, 28, 29]. However, their translation has been underwhelming, largely because macrophage dynamics cannot be monitored in patients [30]. Reliable in vivo visualization would enable rational therapy optimization, evaluation of biological responses, and identification of patients most likely to benefit.

A defining biochemical hallmark of M2‐to‐M1 reprogramming is the elevated production of nitric oxide (NO), which makes it a powerful endogenous biomarker of macrophage functional state [31, 32, 33]. However, conventional NO probes emit in the visible spectrum, where shallow penetration, tissue autofluorescence, and strong absorption severely limit in vivo utility. By contrast, secondary near‐infrared (NIR‐II; 1000–1700 nm) fluorescence offers deep tissue penetration, minimal autofluorescence, and a high signal‐to‐noise ratio (SNR). Thus, NO‐responsive NIR‐II probes provide a unique opportunity to couple macrophage‐targeted immunotherapy with real‐time immune monitoring at clinically relevant depths, bridging the critical gap between experimental success and clinical translation.

Even when M2‐like TAMs are successfully repolarized, their anti‐tumor activity is curtailed by tumor‐intrinsic immune evasion mechanisms [34]. Chief among these is CD47, a broadly expressed “don't eat me” signal that engages SIRPα on macrophages to suppress phagocytosis [35, 36]. CD47 expression is frequently upregulated in TNBC and predicts therapeutic resistance and poor prognosis. These findings highlight the necessity of a dual approach: relieving CD47‐SIRPα checkpoint inhibition while reprogramming TAMs, thereby restoring both surveillance and effector functions of macrophages [37].

**SCHEME 1:**
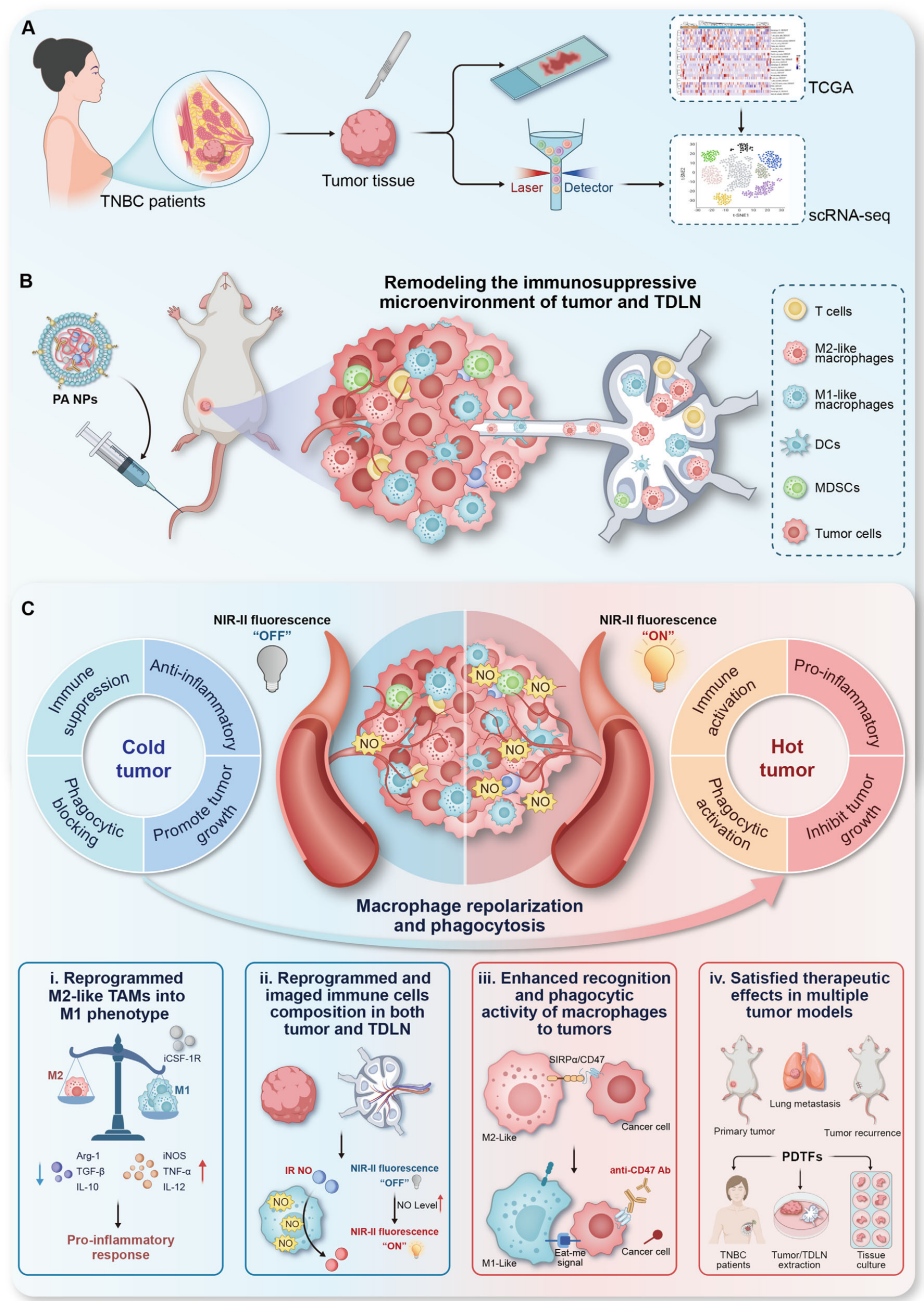
Schematic illustration of biomimetic nanoparticles (PA NPs) for macrophage‐mediated immunotherapy. (A) Procedure for specimen acquisition and data processing in this study. (B) Scheme of tumor microenvironment (TME) remodeling in primary tumor and tumor‐draining lymph nodes (TDLNs). (C) Schematic illustration of anti‐tumor mechanisms and macrophage‐mediated second near‐infrared (NIR‐II) imaging.

In this study, we report TME‐responsive NIR‐II biomimetic nanoparticles (PA NPs) that integrate real‐time immune monitoring with dual macrophage–targeted immunotherapy (Scheme 1). These nanoparticles feature an NO‐activated NIR‐II “off–on” switch, enabling spatiotemporal visualization of TAM polarization and migration in vivo. In parallel, PA NPs were co‐delivered with a CSF‐1R inhibitor to repolarize M2‐like TAMs into tumoricidal M1 phenotypes and an anti‐CD47 antibody to relieve checkpoint‐mediated phagocytic suppression. By targeting both primary tumors and TDLNs, PA NPs dismantle their immunosuppressive interplay and achieve durable tumor regression in primary, metastatic, and recurrent TNBC models and patient‐derived specimens. This study establishes a theranostic paradigm in which immune monitoring and immunomodulation are unified within a single nanoplatform. By simultaneously illuminating macrophage dynamics and reprogramming their function, PA NPs not only potentiate the efficacy of immunotherapy but also lay the foundation for real‐time, personalized monitoring of therapeutic responses in the clinic.

## 2 Results and Discussion

### 2.1 Immune Landscape of Patients with TNBC

**FIGURE 1:**
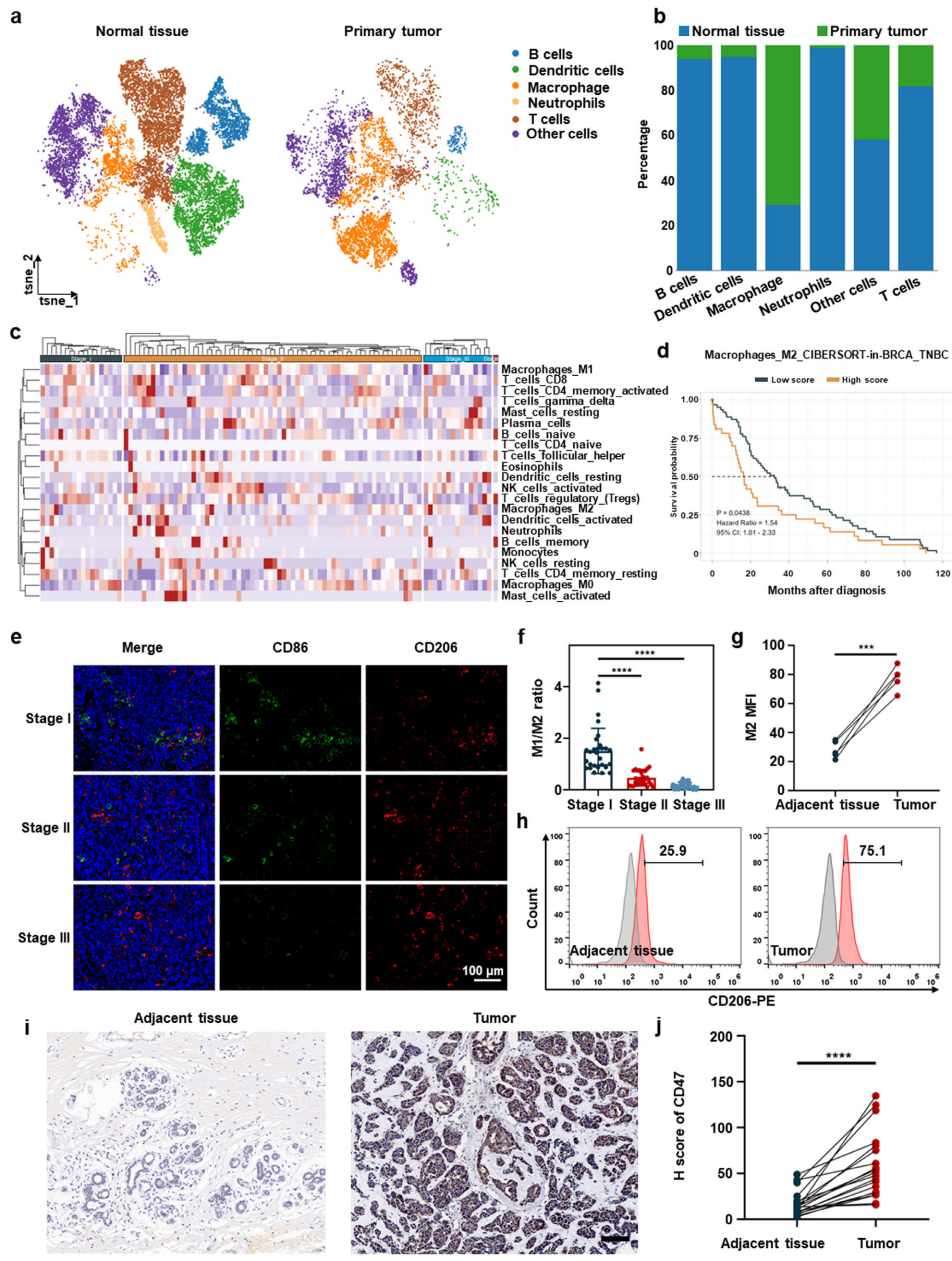
Immune landscape and prognostic significance of triple‐negative breast cancer (TNBC). (a) scRNA‐seq in fresh tissues collected from patients with TNBC (n = 3). (b) Enrichment of immune cells in normal and tumor tissues using scRNA‐seq data. (c) Heatmap depicting the mean normalized abundance of immune cell types detected in stage I, stage II, and stage III samples in the TCGA‐BRCA cohort (n = 101). (d) Kaplan–Meier curves of overall survival according to M2‐like macrophage in patients with TNBC (n = 101). (e) Immunofluorescence staining of M2‐like tumor‐associated macrophages (TAMs) (CD206) and M1‐like TAMs (CD86) in patients with stages I–III TNBC (n = 20); the scale bar is 100 µm. (f) Quantification and statistical analysis of M1/M2 ratio across various stages of patients with TNBC. (g,h) Representative images of flow cytometry of CD206 (M2‐like phenotype) in resected human breast cancer tissue (n = 5). (i,j) Representative CD47 immunofluorescence staining images in patients with breast cancer and H‐score analysis of CD47 expression (n = 20); the scale bar is 100 µm. Statistical significance was determined using one‐way ANOVA with Tukey's multiple comparison tests in (f), paired two‐tailed t‐test in (g) and (j). Data are shown as mean ± SD (*** *p* < 0.001, **** *p* < 0.0001).

The infiltration of immune cells was systematically quantified in patients with TNBC, revealing a pronounced increase in macrophage density within tumor tissues compared with adjacent normal tissues (Figure 1a,b; Figure S1). To further elucidate the dynamics of immune cell populations, we analyzed publicly available datasets to assess the variations in immune cell composition across different clinical stages of TNBC. Strikingly, we observed a robust positive correlation between the abundance of M2‐like macrophages and advanced clinical stages, with higher levels of M2‐like macrophages strongly associated with poorer clinical outcomes (Figure 1c,d). These findings were corroborated by immunofluorescence staining, which confirmed a stage‐dependent increase in M2‐like macrophage infiltration into the tumor tissues (Figure 1e,f). In a complementary analysis, flow cytometry was used to quantify TAM infiltration in a cohort of human breast cancer samples, revealing a significant correlation between the presence of CD206^+^ macrophages and their enrichment in tumor tissues (Figure 1g,h). Furthermore, CD47 expression analysis of tumor sections demonstrated a marked increase in H‐scores relative to adjacent normal tissues (Figure 1i,j). Consistently, single‐cell RNA sequencing analysis revealed a robust upregulation of CD47 expression in tumor tissues compared with that in normal tissues (Figure S2). Collectively, these data highlighted a prominent shift toward M2‐like macrophage polarization coupled with diminished phagocytic function in the TME of breast cancer. This dual mechanism of immune suppression is associated with advanced disease and unfavorable prognosis, underscoring the critical role of functional reprogramming of macrophages in tumor progression. Consequently, therapeutic strategies aimed at reversing M2 polarization and restoring phagocytic activity may offer promising avenues to improve treatment outcomes.

### 2.2 Characteristics of PA NPs

**FIGURE 2:**
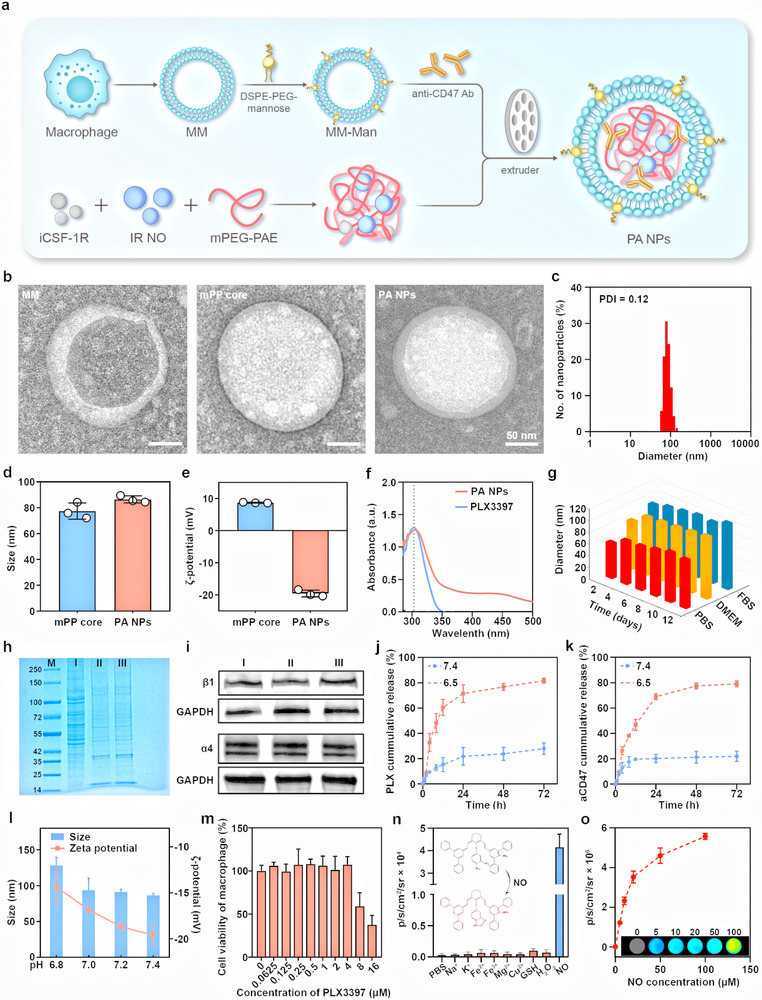
Morphology and characterization of biomimetic nanoparticles (PA NPs). (a) Synthesis process of PA NPs. (b) Transmission electron microscopy images of macrophage membrane (MM), mPEG‐PAE micelles (mPP core), and PA NPs; the scale bar is 50 nm. (c) Size distribution of PA NPs. (d,e) Size and zeta potentials of mPP core and PA NPs (*n* = 3). (f) UV–vis spectrophotometer of PA NPs and free PLX3397. (g) Size stability test of PA NPs in PBS (pH = 7.4), DMEM, and FBS for up to 12 days. (h) Coomassie blue staining of PA NPs. M: Marker, I: Raw264.7 macrophage, II: MM, III: PA NPs. (i) Representative immunoblots of β1 and α4. (j,k) Release profile of PLX3397 and aCD47 at various pH conditions (*n* = 3). (l) Size and zeta potentials of PA NPs at various pH conditions (*n* = 3). (m) Viability of Raw264.7 macrophage after PA NP treatments at different PLX3397 concentrations. (n) Second near‐infrared (NIR‐II) signal intensity of PA NPs treated with PBS or various analytes (100 µm) at 37°C for 15 min. (o) NIR‐II signal intensity of PA NPs treated with various concentrations of nitric oxide (NO). Data are shown as mean ± SD.

To construct PA NPs, we began by extracting the membranes of RAW264.7 macrophages to create biomimetic nanovesicles, leveraging their inherent tumor tropism and biocompatibility (Figure 2a) [38, 39, 40, 41, 42]. The tumoral acidic pH–responsive mPEG‐PAE block copolymer was chosen for its ability to undergo micellization/demicellization transitions between pH 6.4 and 6.8 (Figure S3) while forming a stable self‐assembled polymeric micelle structure at physiological pH 7.4 [43]. We employed a preliminary approach combining macrophage membrane (MM) camouflage with encapsulated mPEG‐PAE micelles (mPP core), iCSF‐1R (PLX3397), and aCD47 using the SFTE method (sonication, freeze‐thawing cycles, and extrusion) [44, 45]. Transmission electron microscopy revealed that the synthesized nanovesicles exhibited a spherical morphology (Figure 2b; Figure S4). Dynamic light scattering (DLS) analysis showed that the PA NPs had an average hydrodynamic diameter of 86.48 ± 2.2 nm (Figure 2c), slightly larger than that of the mPP core (77.4 ± 5.1 nm; Figure 2d), with a negative zeta potential of −19.6 ± 0.86 mV (Figure 2e). The encapsulation efficiency (EE) and loading capacity (LC) of PLX3397 were determined via UV–vis spectrophotometry at 303 nm, yielding an EE of 91.8% ± 0.2% and LC of 5.7% ± 0.01% (Figure 2f). And the EE and LC of aCD47 were determined by fluorescence intensity of Alexa647 labeled aCD47 antibody, yielding an EE of 55.8% ± 1.6% and LC of 1.7% ± 0.05% (Table S1). Importantly, the PA NPs maintained a stable size for 14 days in PBS (pH 7.4), DMEM, and DMEM containing 10% fetal bovine serum (FBS), indicating their stability in biological media (Figure 2g). DSPE–PEG–Mannose was incorporated into PA NPs to enhance the targeting of the mannose receptor (CD206), which is highly expressed on M2‐like TAMs. Surface modification with DSPE–PEG–Mannose was confirmed by fluorescence quantification, with a modification efficiency of 71.4% ± 3.5%. Mannose modification also enhanced the cellular uptake of PA NPs by M2‐like macrophages (Figure S5).

Sodium dodecyl sulfate–polyacrylamide gel electrophoresis (SDS‐PAGE) was performed to verify the successful incorporation of MM proteins onto the PA NPs (Figure 2h). The PA NPs lane (III) clearly exhibited membrane protein bands, confirming the presence of MM proteins on the surface [44]. Specifically, β1 and α4 integrins, which mediate macrophage adhesion and are critical for tumor‐targeting, were identified on the MM and likely contribute to the PA NPs’ tumor‐homing efficiency [46]. Western blot analysis further demonstrated that the levels of β1 and α4 integrins in the PA NPs closely resembled those in the MM and macrophage samples (Figure 2i), suggesting that the fabrication process had minimal impact on these key proteins. Next, the in vitro release profiles of PLX3397 and aCD47 from PA NPs were evaluated under different pH conditions (pH 6.5 and 7.4) using a dialysis method to validate the acid‐responsive dissociation behavior. At physiological pH (pH 7.4), less than 30% of PLX3397 or aCD47 was released over 72 h, whereas at acidic pH (pH 6.5), 81.7% and 79.0% of PLX3397 and aCD47 were released, respectively (Figure 2j,k). This pH‐dependent release behavior, driven by the proton sponge effect, ensures minimal off‐target release under physiological conditions, while maximizing release in acidic tumor environments. Additionally, DLS analysis monitored the pH‐dependent changes in size and zeta potential, confirming the stability and responsiveness of the PA NPs (Figure 2l).

The cytotoxicity of PA NPs was assessed on RAW264.7 cells using the CCK‐8 assay. Significant cytotoxicity was observed only when PLX3397 concentrations exceeded 8 µm (Figure 2m), consistent with macrophage depletion via CSF‐1R pathway inhibition [26]. Further cytotoxicity studies in NIH‐3T3 and HC11 cells (Figures S6 and S7) demonstrated that the PA NPs exhibited excellent cytocompatibility at low PLX3397 concentrations, confirming their safety profile for potential therapeutic applications. To evaluate the potential of PA NPs for NO detection in vivo, we assessed their selective responses to various analytes (Na^+^, K^+^, Fe^2+^, Fe^3+^, Mg^2+^, Cu^2+^, GSH, H_2_O_2_, and NO) (Figure 2n). After incubation with these analytes, NIR‐II fluorescence signals were measured. Notably, only NO induced a significant increase in NIR‐II fluorescence, highlighting the high selectivity of the PA NPs for NO detection (Figure 2o).

To investigate the cellular internalization mechanism of the PA NPs, we used the endocytosis inhibitors such as filipin, chlorpromazine, and dynasore to inhibit caveolae‐, clathrin‐, and actin‐mediated endocytosis. As shown in Figure S8, the cellular uptake efficacies of the PA NPs were seldom affected by the three inhibitors. The membrane fusion pathway of PA NPs was further confirmed using confocal laser scanning microscopy (CLSM). The DiI‐labeled mPP core was loaded onto DiO‐labeled MM to imitate the membrane fusion process. The red signal gradually merged with the green circle, indicating that the PA NPs had fused to the cell membrane [47, 48]. The results consistently demonstrate that PA NPs enter cells primarily via membrane fusion rather than via endocytosis.

### 2.3 PA NPs Reprogram TAMs to M1‐Like Phenotype and Enhance Phagocytosis In Vitro

**FIGURE 3:**
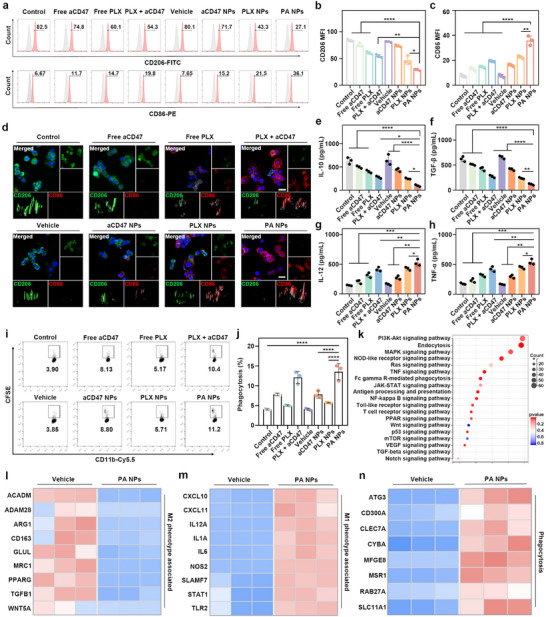
Biomimetic nanoparticles (PA NPs) reprogram macrophage toward the pro‐inflammatory phenotype and enhance macrophage phagocytosis. (a–c) Representative images of flow cytometry and quantification of the expression of CD206 (M2 phenotype) and CD86 (M1 phenotype) in bone marrow‐derived macrophages (BMDMs) after different treatments (n = 3). (d) Confocal laser scanning microscopy (CLSM) imaging shows the changes of macrophage phenotypes in M2 BMDMs after different treatment. The M2‐like macrophages, M1‐like macrophages, and nucleus were stained with CD206 (green), CD86 (red), and DAPI (blue); the scale bar is 20 µm. (e–h) ELISA analysis of the anti‐inflammatory IL‐10, TGF‐β, IL‐12, and TNF‐α after different treatments (n = 3). (i,j) Representative images of flow cytometry and quantification of BMDM phagocytose tumor cells (n = 3). (k) Signaling pathway enrichment analysis of Kyoto Encyclopedia of Genes and Genomes (KEGG) in PA NP–treated BMDMs. (l–n) Expression of selected genes related to M2‐like phenotype, M1‐like phenotype, and phagocytosis in PA NP–treated BMDMs (n = 3). All statistical significance was determined using one‐way ANOVA with Tukey's multiple comparison tests. Data are shown as mean ± SD (* *p* < 0.05, ** *p* < 0.01, *** *p* < 0.001, **** *p* < 0.0001).

To explore the potential of PA NPs to reprogram M2‐like macrophages to an M1‐like phenotype, bone marrow–derived macrophages (BMDMs) were isolated from mice and polarized into an M2‐like phenotype using interleukin‐4 (IL‐4) and various formulations. Flow cytometry and CLSM analyses demonstrated that PA NP treatment significantly reduced the expression of the M2‐like macrophage marker CD206 while inducing the expression of the M1 marker CD86 (Figure 3a–d). The M1/M2‐like macrophage ratio was significantly increased, highlighting the ability of PA NPs to reprogram M2‐like TAMs toward an anti‐tumor M1‐like phenotype (Figure S9). ELISA analysis further corroborated these findings, revealing a marked decrease in M2‐associated cytokines, including interleukin‐10 (IL‐10) and transforming growth factor‐β (TGF‐β), whereas interleukin‐12 (IL‐12) and tumor necrosis factor‐α (TNF‐α)—markers associated with the M1 phenotype—were significantly elevated in the PA NP group compared with those in PLX3397 NPs or aCD47 NPs alone (Figure 3e–h).

To assess the enhanced phagocytic capacity, BMDMs and 4T1 cells were stained with anti‐CD11b‐Cy5.5 and carboxyfluorescein diacetate succinimidyl ester (CFSE), respectively. Flow cytometry analysis of double‐positive (Cy5.5^+^CFSE^+^) cell populations revealed that macrophages treated with PA NPs exhibited a 3.3‐fold increase in 4T1 cell phagocytosis compared with that in vehicle‐treated BMDMs (Figure 3i,j), demonstrating the enhanced phagocytic potential of PA NP–treated macrophages. To gain deeper insights into the effects of PA NPs on macrophage reprogramming and phagocytic enhancement, we performed RNA sequencing analysis of IL‐4–treated BMDMs treated with either vehicle or PA NPs. Principal component analysis clearly separated the transcriptomic profiles of the two groups, highlighting significant differences in gene expression (Figure S10). The distribution of differentially expressed genes was visualized (Figure S11), confirming the substantial impact of PA NPs on macrophage gene expression.

Kyoto Encyclopedia of Genes and Genomes (KEGG) analysis further revealed that PA NPs regulated gene expression through key pathways such as the PI3K–Akt signaling pathway, endocytosis, and FcγR‐mediated phagocytosis (Figure 3k). Additionally, PA NP treatment enhanced the anti‐tumor functions of macrophages, including M1‐like phenotype polarization, inflammatory response activation, antigen processing and presentation, and phagocytosis, while simultaneously suppressing protumor functions such as M2‐like polarization and inflammatory response inhibition (Figure 3l–n). These bioinformatic results suggest that PA NPs can effectively reprogram macrophages by inhibiting M2 polarization signaling pathways and enhancing tumor cell phagocytosis, thereby promoting anti‐tumor immunity. Collectively, our study introduces a distinct immunotherapeutic paradigm by integrating TAM reprogramming with anti‐CD47 therapy and real‐time imaging. Unlike existing TAM‐targeted strategies such as CSF‐1R inhibitors, which primarily drive macrophage reprogramming but fail to address tumor‐intrinsic “don't eat me” signals, our PA NPs uniquely combine a CSF‐1R inhibitor with anti‐CD47 therapy. This dual‐action approach not only reprograms immunosuppressive TAMs but also actively restores their phagocytic capacity against tumor cells, thereby overcoming a key limitation of CSF‐1R monotherapy. This synergistic combination of therapeutic and diagnostic functions within a single nanoplatform provides a more comprehensive and therapeutically potent alternative to conventional TAM‐targeted therapies.

### 2.4 Pharmacokinetics, Biodistribution, and NO‐Responsive NIR‐II Imaging of PA NPs

The in vivo circulation behavior of PA NPs was evaluated in male Sprague–Dawley (SD) rats by intravenous injection of IR825‐labeled PA NPs. Blood samples were collected at predefined time intervals, and IR825 fluorescence intensity was detected. As shown in Figure S12, a longer circulation time (t_1/2_ = 9.68 h) was observed in the PA NP groups than in the free IR825 group (t_1/2_ = 4.37 h), along with a higher area under the curve and lower clearance rates (Table S2) [49, 50].

**FIGURE 4:**
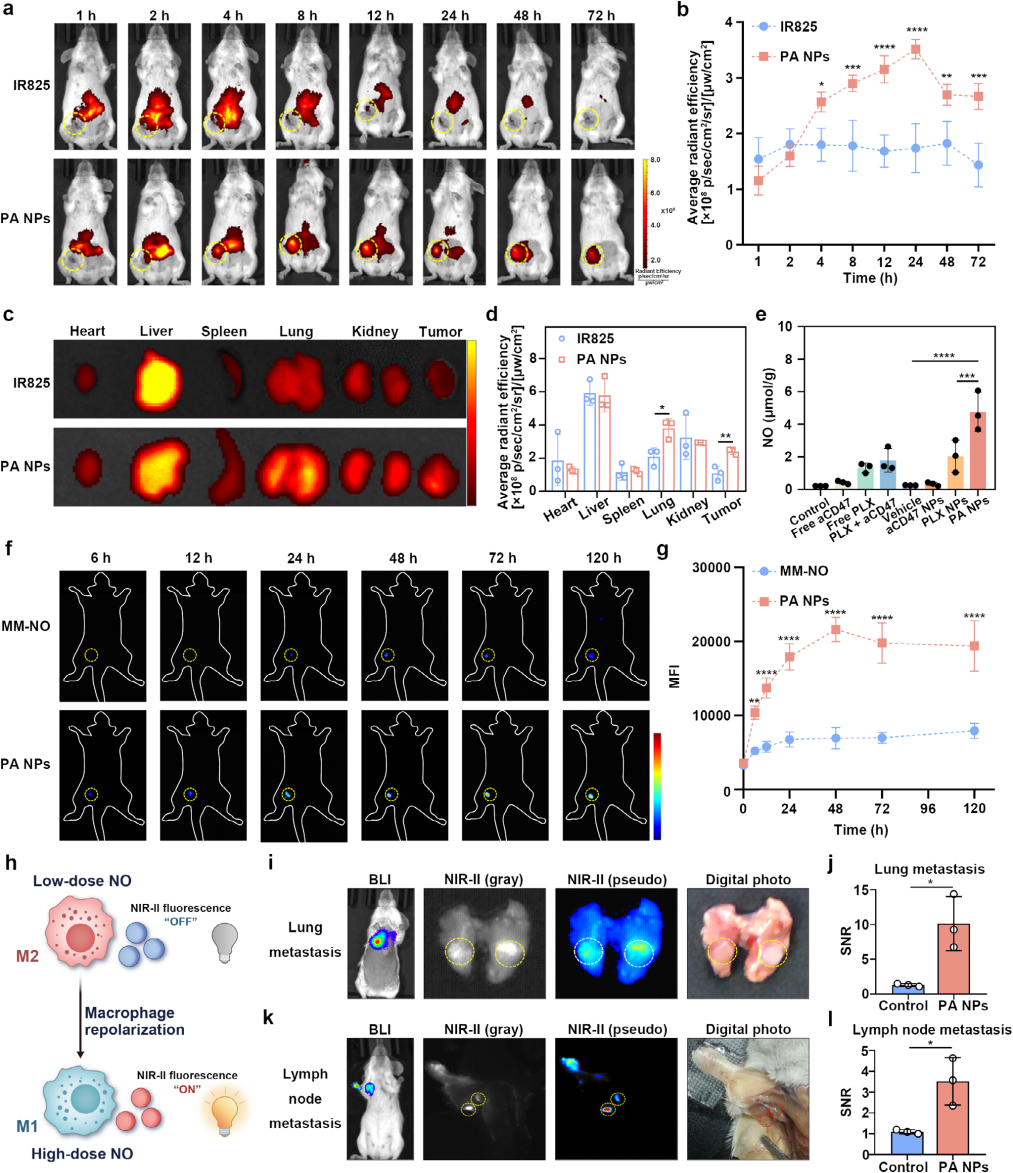
Biodistribution and second near‐infrared (NIR‐II) images in primary tumor and metastasis models. (a,b) In vivo fluorescence imaging of 4T1 tumor–bearing mice at a series of time points after intravenous injection of IR825, and IR825‐labeled PA NPs (*n* = 3). (c,d) Ex vivo fluorescence imaging of tumors and major organs at 72 h post‐injection (*n* = 3). (e) Nitric oxide (NO) concentration of tumor after different treatment (*n* = 3). (f,g) Time‐course NIR‐II images and corresponding quantified mean fluorescence intensity at tumor region (*n* = 3). (h) Schematic illustration of macrophage reprogramming and NO responsive NIR‐II imaging. (i) Representative bioluminescence image (BLI) and NIR‐II and bright‐field images of 4T1‐luc lung metastatic mice at 48 h after intravenous injection with PA NPs. (j) Signal‐to‐noise ratio (SNR) of lung metastatic tumors after different treatment (*n* = 3). (k) Representative BLI and NIR‐II and bright‐field images of 4T1‐luc lymph node metastatic mice at 48 h after intravenous injection with PA NPs. (l) SNR of lymph node metastatic tumors after different treatment (*n* = 3). Statistical significance was determined using unpaired two‐tailed *t*‐test in (d), (j), and (l), one‐way ANOVA with Tukey's multiple comparison tests in (e), Sidak's multiple comparisons test in (b) and (g). Data are shown as mean ± SD (^*^ *p* < 0.05, ^**^ *p* < 0.01, ^***^ *p* < 0.001, ^****^ *p* < 0.0001).

The biodistribution of PA NPs was assessed in 4T1 breast tumor models following the intravenous administration of IR825‐labeled PA NPs or free IR825 (Figure 4a). Fluorescence imaging revealed significantly stronger tumor‐specific signals in mice treated with PA NPs than in those treated with free IR825, with peak signal intensity observed 24 h post‐injection (Figure 4b). Notably, PA NP–treated mice maintained strong fluorescence signals in tumors even at 72 h, whereas the IR825‐treated group exhibited consistently low tumor fluorescence. This result underscores the superior tumor‐targeting ability and in vivo accumulation of the PA NPs. Imaging of major organs and tumors 72 h after administration further validated the enhanced tumor accumulation and prolonged retention of PA NPs (Figure 4c,d).

To quantify the NO concentration in vivo, 4T1 tumor–bearing mice were administered various treatments, and tumor tissues were excised for NO quantification using a colorimetric assay kit (Figure 4e). PA NP–treated tumors exhibited the highest NO concentration 72 h after intravenous injection, indicating the effective targeting of M2‐like macrophages and enhanced NO release within the TME.

In vivo NIR‐II fluorescence imaging was performed to monitor the targeting and polarization of M2‐like macrophages using the PA NPs in orthotopic 4T1 tumors, lymph node metastasis, and lung metastatic tumor models. In the orthotopic breast cancer model, female BALB/c mice received intravenous injections of either PA NPs or PLX3397 (without nanovesicles, MM‐NO), and time‐course NIR‐II images were captured at 1050 nm under 808 nm laser excitation. As shown in Figure 4f, the fluorescence signal in the tumor region displayed a time‐dependent increase over 48 h post‐injection of PA NPs, reaching a plateau with a 3.1‐fold enhancement in signal intensity compared with that in the MM‐NO group (Figure 4g). This finding indicates that PA NPs effectively utilize NO secreted by M1‐like macrophages in the TME to enhance the fluorescence signals (Figure 4h). Future iterations should incorporate several refinements to enhance the specificity of M2 TAM targeting. These include dual‐targeting approaches that combine M2‐specific moieties with tumor‐homing properties. Additionally, M2‐responsive release mechanisms triggered by microenvironmental cues unique to M2 TAMs can be employed. Furthermore, the use of more selective inhibitors or antibody‐drug conjugates could help reduce off‐target effects. Combined with the pH‐responsive and biomimetic properties of the PA NPs, these refinements have the potential to broaden the therapeutic window and facilitate clinical translation [51, 52].

M2‐like macrophages play a critical role in tumor growth and metastasis. To evaluate the accumulation of PA NPs in metastatic lesions, in vivo NIR‐II fluorescence imaging was performed in a lung metastasis model following intravenous administration (Figure 4i). Representative bioluminescence images (BLIs) were acquired using the IVIS system to confirm the successful establishment of the model, with metastatic tumors observed 14 days after the intravenous injection of 4T1‐luc tumor cells (Figure 4i). After 48 h, the metastatic lung tumors exhibited clear fluorescence signals in ex vivo lung NIR‐II fluorescence images, demonstrating the enhanced sensitivity of PA NPs. Furthermore, a high SNR of 10 was achieved for metastatic tumors, suggesting its potential for imaging‐guided surgery and other clinical applications (Figure 4j).

Finally, in vivo NIR‐II fluorescence imaging of metastatic tumors in the lymph nodes was performed after the intravenous administration of PA NPs (Figure 4k). The BLIs clearly indicated the presence of metastatic 4T1‐luc tumor cells in the lymph nodes near the footpad injection site. Following the PA NPs injection, the lymph nodes were illuminated at 48 h, showing a high SNR (Figure 4l).

### 2.5 Anti‐Tumor Efficacy and Local Immune Responses by PA NPs In Vivo

To assess the biosafety of PA NPs in vivo, hematological examinations were performed, revealing significant differences in hematological toxicity biochemical parameters across the treatment groups (Figures S13 and S14). Compared with the control group, the PA NP group showed no significant decline in red blood cells or platelets, whereas free aCD47 treatment caused significant blood toxicity within 1 week after intravenous administration. Collectively, these results demonstrate the superior tumor‐targeting capability, prolonged retention, and excellent biocompatibility of PA NPs, underscoring their potential for safe and effective in vivo applications.

**FIGURE 5:**
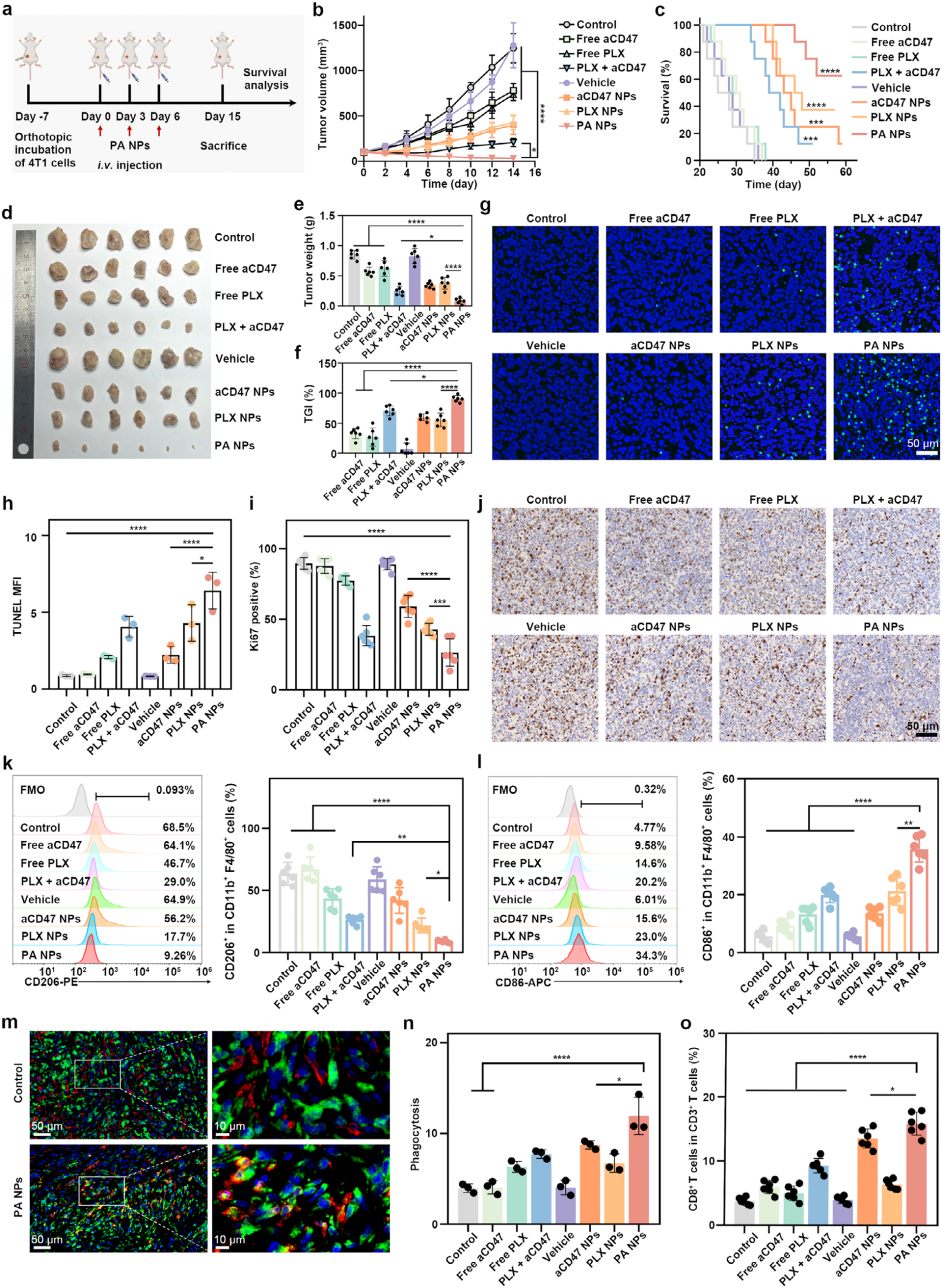
Anti‐tumor effects against 4T1 primary tumor model. (a) Schematic illustration of the experimental schedule. (b) Tumor growth profiles of primary tumors in 4T1 orthotopic tumor–bearing mice after different treatments (*n* = 6). (c) Survival curves of mice after different treatments (*n* = 8). (d) Photograph of tumors excised from 4T1 orthotopic tumor–bearing mice after different treatments on day 14 (*n* = 6). (e) Weight of tumors excised from mice on day 14 (*n* = 6). (f) Tumor growth inhibition of 4T1 orthotopic tumor–bearing mice after different treatments (*n* = 6). (g,h) Representative images and quantitative analysis of TUNEL staining of tumor sections after different treatments (*n* = 3); the scale bar is 50 µm. (i,j) Representative images and quantitative analysis of Ki67 staining of tumor sections after different treatments (*n* = 6); the scale bar is 50 µm. (k,l) Representative flow cytometry histograms and the quantitative analysis of the expression of CD206 (k) and CD86 (l) in tumors after different treatments (*n* = 6). (m) Representative immunofluorescence images of 4T1‐GFP tumor tissues stained for tumor cells (GFP, green), macrophages (F4/80, red), and nuclei (DAPI, blue). (n) Quantitative analysis of macrophage (F4/80) phagocytosis of 4T1‐GFP tumor cells after different treatments (*n* = 3). (o) Quantitative analysis of tumor‐infiltrating CD8^+^ in CD3^+^ T cells after different treatments (*n* = 6). Statistical significance was determined using log‐rank test in (c), one‐way ANOVA with Tukey's multiple comparison tests in (b), (e,f), (h,i), (k,l), and (n,o). Data are shown as mean ± SD (^*^ *p* < 0.05, ^**^ *p* < 0.01, ^***^ *p* < 0.001, ^****^ *p* < 0.0001).

The therapeutic efficacy of the PA NPs was evaluated in 4T1 tumor–bearing mice according to the treatment schedule outlined in Figure 5a. Tumor volumes in the free aCD47 and free PLX groups increased rapidly during treatment, whereas PA NPs significantly suppressed tumor growth compared with that in all other treatment groups (Figure 5b). In a long‐term survival study, mice treated with PA NPs demonstrated a 60% survival rate over a 60‐day observation period, significantly outperforming other treatments (Figure 5c). Tumors were excised, imaged, and weighed to further assess the treatment efficacy (Figure 5d). The tumor weights in the PA NP group were reduced to approximately 9.5% of those in the control group (Figure 5e,f). Histological analyses, including TUNEL staining (Figure 5g,h) and Ki67 immunohistochemistry (Figure 5i,j), along with confirmation by hematoxylin and eosin (H&E) staining and CD31 immunohistochemistry (Figures S15 and S16), provided evidence of treatment efficacy. H&E staining revealed extensive tumor necrosis in the PA NP group. Ki67 and CD31 expression levels were significantly reduced, indicating suppressed cell proliferation and angiogenesis. TUNEL staining revealed markedly increased tumor apoptosis in the PA NP group. These results were further confirmed in EMT6 tumor–bearing mice, where PA NP treatment led to complete tumor remission in 75% of the treated mice and significantly improved survival (Figures S17 and S18). Tumor photographs, weight measurements, and tumor growth inhibition further demonstrated superior tumor regression effects (Figures S19–S21). The anti‐tumor activity of the PA NPs was further evidenced by a significant reduction in Ki67 expression, indicating suppressed tumor proliferation (Figures S22 and S23). Additionally, TUNEL staining confirmed a markedly increased rate of apoptosis (Figures S24 and S25). These findings underscore the robust anti‐tumor efficacy of the PA NPs in orthotopic breast cancer models.

Inspired by the excellent therapeutic efficacy of PA NPs in vivo, we evaluated their macrophage polarization capacity in a tumor‐immunosuppressive environment using a 4T1‐bearing mouse model. To elucidate the anti‐tumor immune mechanisms triggered by PA NPs, the percentage of various immune cells in primary tumor tissues was assessed via flow cytometry. Additionally, typical immune cell cytokines in tumor tissues following different treatments were measured using ELISA. As anticipated, the potential of PA NPs to re‐educate TAMs into M1‐like phenotypes was further explored in vivo. The population of M2‐like TAMs in the tumors decreased significantly from 63.5% in the control group to 9.0% in the PA NP group (Figure 5k). Consistently, PA NPs markedly increased the number of M1‐like TAMs, which was 5.5‐, 0.8‐, 1.6‐, and 0.6‐fold higher than that in the vehicle, free aCD47 + PLX, aCD47 NP, and PLX NP groups, respectively (Figure 5l). Accordingly, the M1/M2 ratio increased approximately 32‐fold in the PA NP group compared with that in the control group, significantly surpassing all other treatment groups (Figure S26). The potential of PA NPs to reprogram M2‐like macrophages into the M1 phenotype was also confirmed in EMT6 tumor–bearing mice (Figures S27–S29). The more pronounced M1/M2 shift observed in 4T1 tumors than in EMT6 tumors likely reflects their distinct baseline immune features. The highly immunosuppressive microenvironment of 4T1 tumors, with abundant baseline M2‐like macrophages, may provide a greater dynamic range for therapeutic repolarization by PA NPs.

Next, we established a green fluorescent protein (GFP)‐transduced 4T1 cell line (4T1‐GFP) to assess whether PA NPs promote the phagocytosis of tumor cells in vivo. Merged immunofluorescence images revealed that PA NP therapy resulted in stronger yellow fluorescence than that observed in the control group, suggesting enhanced macrophage phagocytosis of tumor cells in vivo (Figure 5m). Together with the enhanced phagocytic ability of TAMs through CD47 blockade and the reprogramming of the suppressive immune microenvironment by PA NPs (Figure 5n; Figure S30), the reprogrammed TAMs significantly improved T cell infiltration of primary tumors in mice (Figure 5o; Figure S31). Furthermore, this therapy led to a 4.2‐fold increase in CD8^+^ T cell infiltration within tumors compared with that in the control group (Figures S32 and S33). Intratumoral immune cell infiltration plays a pivotal role in the success of immunotherapy, and our results demonstrated that tumor‐infiltrating CD3^+^CD8^+^ T cells in the PA NP group were significantly higher than those in the vehicle group. Notably, these key findings regarding enhanced phagocytosis and increased CD8^+^ T cell infiltration were further confirmed in the EMT6‐bearing mouse model, underscoring the broad applicability of PA NPs in promoting anti‐tumor immunity (Figures S34 and S35).

Although our data demonstrated that PA NPs combined TAM reprogramming with CD47 blockade to achieve superior anti‐tumor efficacy, we acknowledge that the individual contribution of the CD47 blockade was primarily supported by in vitro phagocytosis assays and comparison with control groups lacking aCD47. Future mechanistic studies employing genetic approaches, such as CD47 knockout tumor cells or SIRPα blocking antibodies in vivo, will be necessary to fully dissect the relative contributions of each pathway. Nonetheless, the synergistic combination strategy presented herein offers a robust therapeutic approach for TNBC that leverages both macrophage repolarization and checkpoint inhibition.

### 2.6 TDLN Immune Microenvironment Reprogrammed by PA NPs in TNBC Mice

**FIGURE 6:**
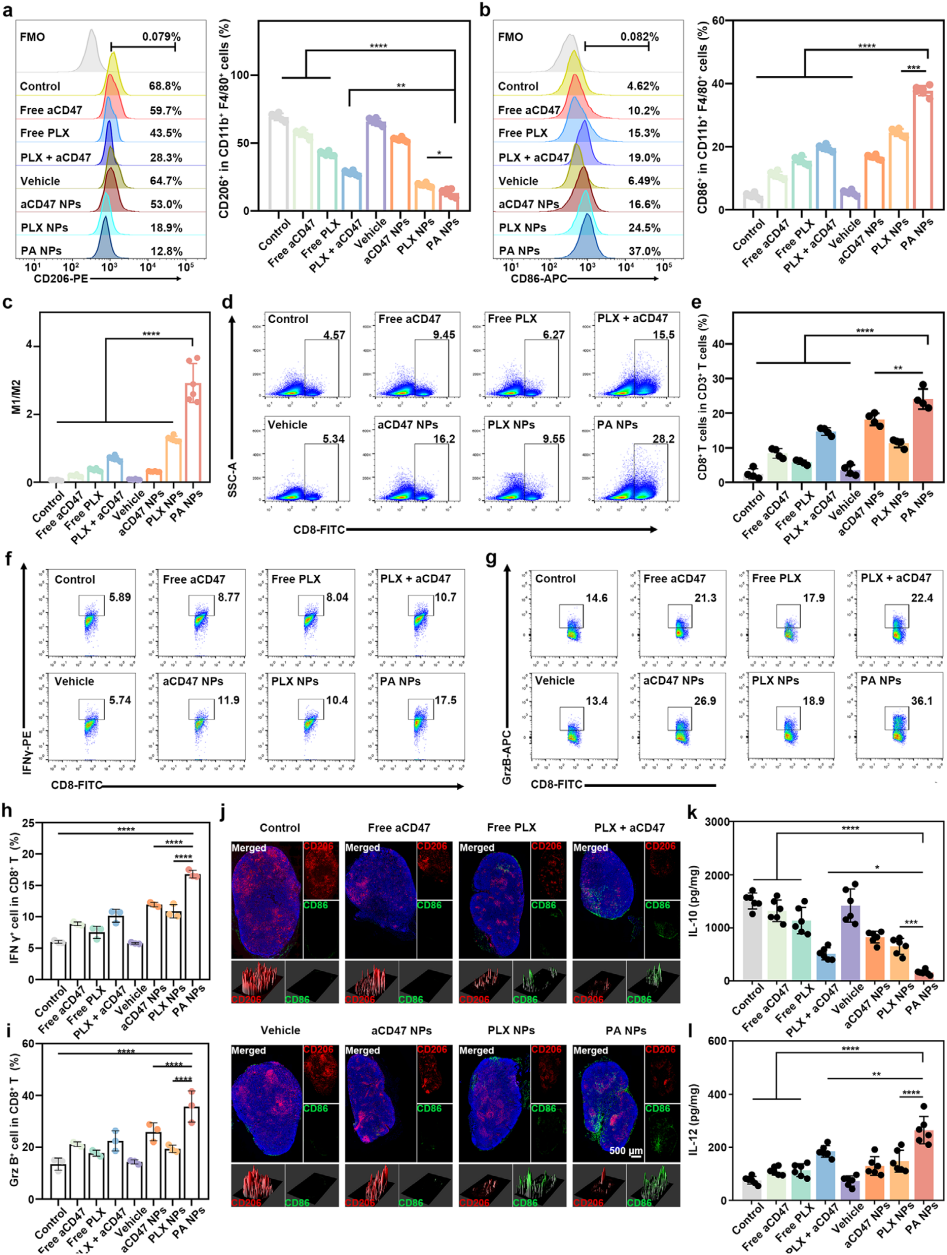
Immune microenvironment reprogramming in lymph nodes of 4T1 tumor model. (a–c) Representative flow cytometry histograms and the quantitative analysis of the expression of CD206 (a), CD86 (b), and M1/M2 ratio (c) in lymph nodes after different treatments (*n* = 6). (d,e) Representative flow cytometry plots and quantitative analysis of CD8^+^ in CD3^+^ T cells in lymph nodes after different treatments (*n* = 4). (f–i) Percentage of IFN‐γ^+^ T cells (f), Granzyme B^+^ T cells (g), and their quantitative analysis (h,i) in 4T1 tumors after different treatments (*n* = 3). (j) Confocal laser scanning microscopy (CLSM) images of tumor‐draining lymph node (TDLN) section after different treatment; the scale bar is 500 µm. (k,l) ELISA analysis of interleukin (IL)‐10 (k) and IL‐12 (l) in TDLNs after different treatments (*n* = 6). All statistical significance was determined using one‐way ANOVA with Tukey's multiple comparison tests in (a–c), (e), (h,i), and (k,l). Data are shown as mean ± SD (^*^ *p* < 0.05, ^**^ *p* < 0.01, ^***^ *p* < 0.001, ^****^ *p* < 0.0001).

To investigate the effect of the PA NPs on the TDLN immune microenvironment, we assessed their ability to modulate TDLNs. Flow cytometry was used to analyze immune cell subpopulation changes in the lymph nodes of 4T1 tumor–bearing mice following treatment. The analysis revealed a 3.9‐ to 1.4‐fold reduction in M2‐like macrophages and a 2.3‐ to 1.5‐fold increase in M1‐like macrophages in the PA NP–treated group compared with those in the aCD47 NP and PLX NP groups (Figure 6a,b). Notably, the M1/M2‐like macrophage ratio in the PA NP–treated group increased by 4.2‐fold compared with that in the free aCD47 + PLX3397 group, further emphasizing the reprogramming potential of PA NPs (Figure 6c).

Additionally, CD3^+^CD8^+^ T cells, which are crucial components of anti‐tumor immunity, were significantly elevated in the TDLNs following PA NP treatment. The proportion of CD3^+^CD8^+^ T cells in the PA NP group was higher than that in the free aCD47 + PLX3397 group and six times greater than that in the vehicle‐treated group (Figure 6d,e). These findings suggest that PA NP treatment can effectively shift TDLNs from an immunosuppressive to an immunoactive state, enhancing localized immune responses against residual tumor metastasis. To further assess the activation of tumor‐specific immune responses, we examined the populations of CD8^+^ IFN‐γ^+^ T and CD8^+^ Granzyme B^+^ T cells, both of which are critical for anti‐tumor immunity. Treatment with free aCD47 + PLX3397 significantly increased the infiltration of activated CD8+ T cells (Figure 6f,g). However, the PA NP treatment induced the highest proportions of CD8^+^ IFN‐γ^+^ T and CD8^+^ Granzyme B^+^ T cells (Figure 6h,i), highlighting the potent immune activation triggered by PA NPs. This enhanced tumor infiltration of cytotoxic T lymphocytes further underscores the immunoregulatory capability of PA NPs.

Furthermore, CLSM images of whole lymph nodes confirmed that PA NP treatment resulted in a notable decrease in M2‐like macrophages and a significant increase in M1‐like macrophages compared with those in the other treatments and vehicle group (Figure 6j). Additionally, PA NP treatment led to significantly increased production of anti‐tumor cytokines, such as IL‐12 and IFN‐γ, and reduced production of protumor cytokines, including IL‐10 and TGF‐β, in the lymph nodes compared with those in the other treatment groups (Figure 6k,l). Collectively, these results demonstrate that PA NPs effectively reprogrammed the immune microenvironment of TDLNs, contributing to enhanced anti‐tumor efficacy in the 4T1 mouse model. Reprogramming of TDLNs from an immunosuppressive to an immunoactive state is crucial for improving the overall anti‐tumor immune response.

### 2.7 Pulmonary Metastasis Suppression and Recurrence Inhibition by PA NPs

**FIGURE 7:**
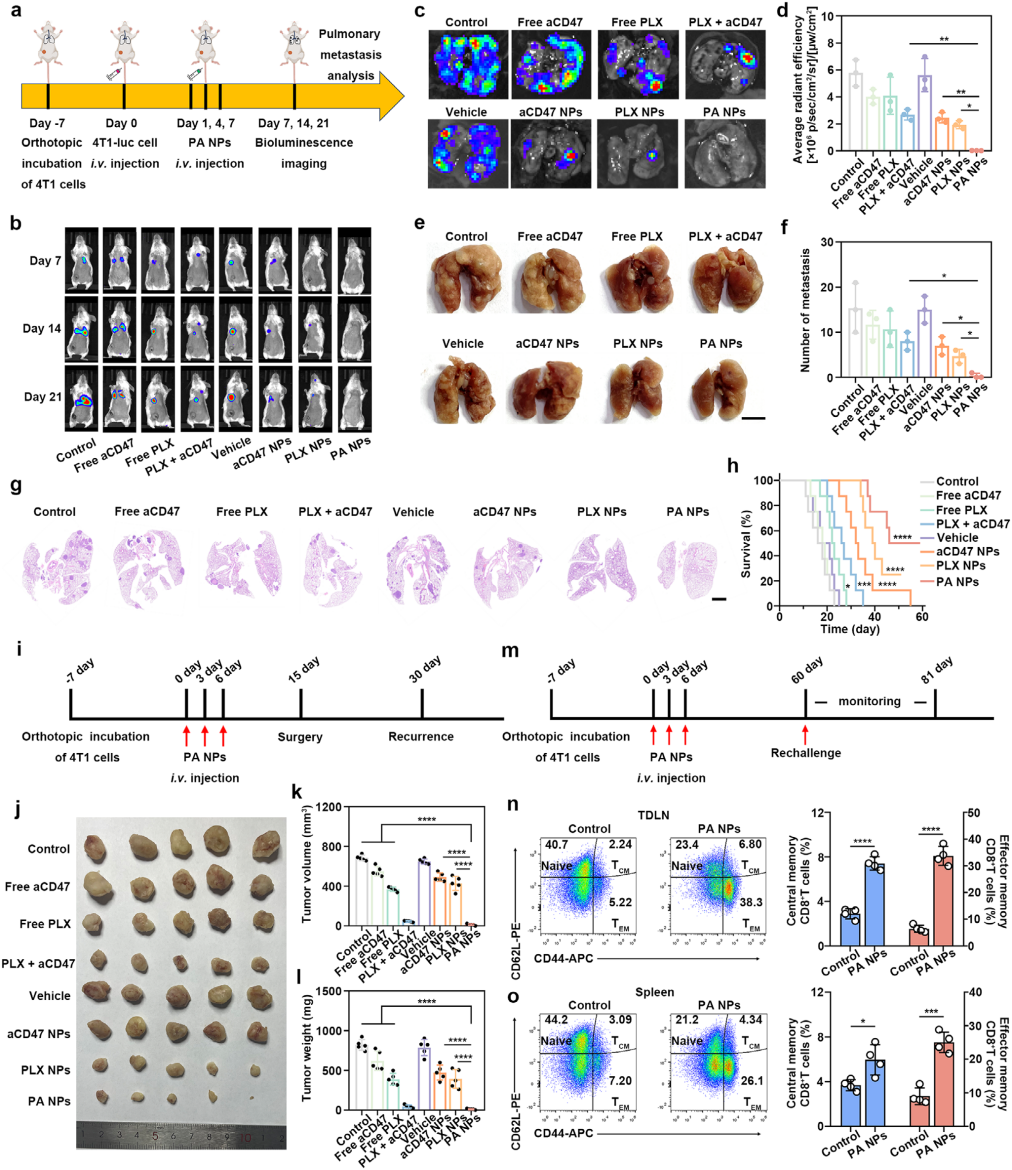
Immune microenvironment reprogramming in lung metastases, recurrent tumor, and rechallenge tumor models. (a) Scheme of lung metastases model experimental design. (b) In vivo bioluminescence imaging of 4T1 lung metastasis models after treatment (*n* = 3). (c,d) Ex vivo bioluminescence imaging (c) of excised lungs and corresponding fluorescence quantitation (d) after different treatments (*n* = 3). (e–g) Photographs (scale bar = 5000 µm), number of lung metastases (f), and hematoxylin and eosin (H&E) staining (g) of excised lungs after different treatments (*n* = 3); the scale bar for H&E staining is 2000 µm. (h) Survival curves of mice with lung metastases (*n* = 8). (i) Schematic of the treatment regimen for the recurrent tumor model. (j–l) Photographs (j), tumor volume (k), and tumor weight (l) of excised recurrent tumors after different treatments (*n* = 5). (m) Schematic illustration of the rechallenge model. (n,o) Representative images of flow cytometry and quantification of central memory (CD44^+^CD62L^+^) and effector memory (CD44^+^CD62L^−^) T cells (CD3^+^CD8^+^) in the tumor‐draining lymph nodes (TDLNs) and spleen (*n* = 4). Statistical significance was determined using unpaired two‐tailed t‐test in (n‐o), log‐rank test in (h), one‐way ANOVA with Tukey's multiple comparison tests in (d), (f), (k‐l). Data are shown as mean ± SD (^*^ *p* < 0.05, ^**^ *p* < 0.01, ^***^ *p* < 0.001, ^****^ *p* < 0.0001).

To further validate the efficacy of PA NPs in inhibiting pulmonary metastasis, primary tumor and pulmonary metastasis models were established by orthotopic implantation of 4T1 cells and intravenous injection of 4T1‐luc cells, respectively (Figure 7a). Lung metastasis was monitored after various treatments. Notably, no visible metastatic lesions were detected in the lungs of the mice treated with PA NPs (Figure 7b). Ex vivo bioluminescence imaging of the lung tissue confirmed the complete absence of metastatic signals in the PA NP–treated group (Figure 7c,d). Furthermore, photographs of lung tissues reinforced these findings, demonstrating the potent anti‐metastatic activity of PA NPs (Figure 7e,f). H&E staining revealed minimal evidence of metastasis in the PA NP–treated group (Figure 7g). These results strongly suggest that PA NPs effectively triggered a robust systemic immune response, leading to the inhibition of pulmonary metastasis. In addition, treatment with PA NPs significantly prolonged survival over the 60‐day observation period (Figure 7h).

To investigate the long‐term immune memory effects of the PA NPs, a recurrent tumor model was established. To prolong survival in 4T1 tumor–bearing mice, primary tumors were surgically removed to 90% of their volume on Day 0, and tumor site recurrence was monitored for 15 days (Figure 7i). The PA NP–treated group exhibited the slowest growth of recurrent tumors compared with that in all other treatment groups (Figure 7j–l), suggesting that PA NPs not only inhibited primary tumor growth but also effectively delayed tumor recurrence. Building on the powerful tumoricidal effects and systemic immune response triggered by PA NPs in tumor remission and recurrence inhibition, we performed tumor rechallenge experiments on Day 60 after PA NP treatment in mice with tumor remission. The 4T1 cells were implanted into the mammary fat pads of these mice without further treatment (Figure 7m). Compared with naïve mice (BALB/c control group), the mice with long‐term tumor remission and prior PA NP treatment exhibited higher frequencies of central memory T cells (CD3^+^CD8^+^CD62L^+^CD44^+^, Tcm) and effector memory T cells (CD3^+^CD8^+^CD62L^−^CD44^+^) in both the TDLNs and spleen (Figure 7n,o). These data suggest that PA NP treatment induces a durable and potent immunological memory, which contributes to the inhibition of tumor metastasis and recurrence in orthotopic breast cancer models.

### 2.8 Patient‐Derived Tumors and TDLN Fragments Validate the Clinical Translational Potential of PA NPs

**FIGURE 8:**
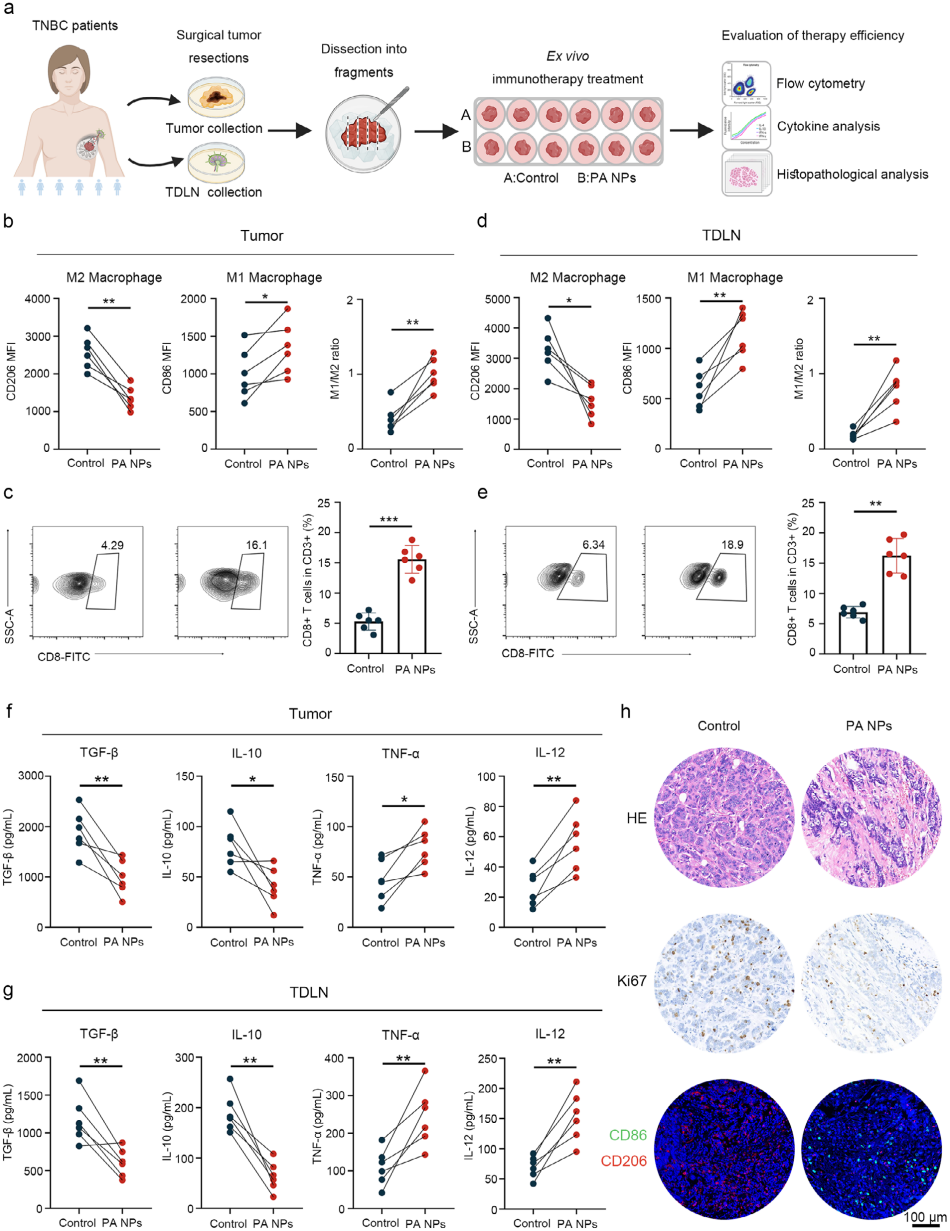
Biomimetic nanoparticles (PA NPs) reprogram the immune microenvironment and exert potent anti‐tumor efficacy in a patient‐derived platform. (a) Schematic diagram illustrating the experimental setup of treating patient‐derived tumor and TDLN fragments with PA NPs. (b) Flow cytometric analysis showing the proportions of M1‐ and M2‐like macrophages and the M1/M2 ratio in treated tumor fragments (n = 6). (c) Frequency of infiltrated CD8^+^ T cells in tumor tissues (n = 6). (d,e) Analysis of macrophage polarization (d) and CD8^+^ T cell infiltration (e) in tumor‐draining lymph nodes (TDLNs) (n = 6). (f,g) Levels of immunosuppressive (TGF‐β and IL‐10) and pro‐inflammatory (TNF‐α and IL‐12) cytokines. (h) Representative histological images of hematoxylin and eosin (H&E) staining, Ki67 immunohistochemistry, and immunofluorescence staining for CD86 (M1 marker, green) and CD206 (M2 marker, red). Scale bar = 100 µm. Statistical significance was determined using paired two‐tailed t‐test in (b), (d), and (f,g), unpaired two‐tailed t‐test in (c) and (e). Data are shown as mean ± SD (* *p* < 0.05, ** *p* < 0.01, *** *p* < 0.001).

To evaluate the anti‐tumor effects and immunomodulatory properties of PA NPs, we established an ex vivo platform using patient‐derived tumors and TDLN tissues. As illustrated in the schematic diagram (Figure 8a), fresh clinical samples were sectioned and randomly assigned to either the PBS or PA NP treatment group to minimize the impact of tissue heterogeneity. Treatment with PA NPs markedly remodeled the tumor immune microenvironment. Flow cytometry analysis revealed that the PA NPs significantly reduced the proportion of M2‐like macrophages and increased the proportion of M1‐like macrophages, leading to a substantially elevated M1/M2 ratio (Figure 8b). Furthermore, PA NP treatment promoted the infiltration of CD8^+^ T cells into the tumor tissues (Figure 8c). A similar immunomodulatory trend was observed in TDLNs, where PA NPs induced macrophage reprogramming toward the M1 phenotype (Figure 8d) and enhanced the infiltration of CD8^+^ T cells (Figure 8e). Cytokine profiling corroborated the immune‐activating effects of PA NPs. In tumor tissues, levels of immunosuppressive cytokines such as TGF‐β and IL‐10 were decreased, whereas those of the pro‐inflammatory cytokines such as TNF‐α and IL‐12 were increased (Figure 8f). Parallel changes were detected in TDLNs, indicating a systemic immunomodulatory response (Figure 8g). Histological and immunofluorescence analyses provided additional evidence of treatment efficacy. H&E staining revealed extensive tumor cell necrosis in the PA NP–treated group. Ki67 expression was notably downregulated, indicating suppressed tumor proliferation. Moreover, immunofluorescence staining confirmed an increase in CD86^+^ M1‐like macrophages and a decrease in CD206^+^ M2‐like macrophages, validating the pro‐inflammatory reprogramming of TAMs (Figure 8h). Collectively, these findings demonstrate that PA NPs effectively reverse immunosuppression in both TNBC tumors and TDLNs, enhance anti‐tumor immunity, and exert potent therapeutic effects, thereby supporting their further clinical development.

## 3 Conclusion

In summary, we developed multifunctional NIR‐II nanoparticles that integrate real‐time imaging with dual immunomodulation to enhance TNBC immunotherapy. By targeting both the TME and TDLNs, PA NPs effectively reprogrammed TAMs, blocked immune evasion signals, and enabled noninvasive monitoring of the treatment response. This strategy not only improves primary tumor control but also suppresses metastasis and recurrence, offering a promising approach to overcome immunosuppressive barriers in TNBC. The therapeutic efficacy of this strategy was demonstrated using a patient‐derived tumor and TDLN fragment. Clinical translation of this technology holds great promise for improving the outcomes of patients with this aggressive breast cancer subtype.

## Funding

This work was supported by National Key R&D Program of China (No.2021YFF0501504, 2021YFA1101304), the National Natural Science Foundation of China (NSFC, No. 82372024, 82402353, 82330060, 92359304, 82427803, 82302274, 82273914), Jiangsu Province Frontier Technology Research and Development Project (No. BF2025609), the China Postdoctoral Science Foundation (2023M740617, 2025T180648), Research Personnel Cultivation Programme of Zhongda Hospital, Southeast University (CZXM‐GSP‐RC120), the Fundamental Research Funds for the Central Universities (‘Zhishan’ Youth Scholar of Southeast University, No. 2242024RCB0056), the Jiangsu Province High‐Level Hospital Pairing Assistance Construction Funds (Zhongda Hospital Affiliated to Southeast University, No.zdlyg20), the Medical Imaging Artificial Intelligence Special Research Fund Project by Nanjing Medical Association Radiology Branch (No.10), Jiangsu Excellent Postdoctoral Program (2024ZB365), SEU Innovation Capability Enhancement Plan for Doctoral Students (CXJH_SEU 24233).

## Conflicts of Interest

The authors declare no conflicts of interest.

## Supporting information




**Supporting File**: advs75593‐sup‐0001‐SuppMat.pdf.

## Data Availability

The data that support the findings of this study are available from the corresponding author upon reasonable request.

## References

[1] A. N. Giaquinto , H. Sung , L. A. Newman , et al., “Breast Cancer Statistics 2024,” CA: A Cancer Journal for Clinicians 74 (2024): 477–495. 39352042 10.3322/caac.21863

[2] N. S. Wagle , L. Nogueira , T. P. Devasia , et al., “Cancer Treatment and Survivorship Statistics, 2025,” CA: A Cancer Journal for Clinicians 75 (2025): 308–340. 40445120 10.3322/caac.70011 PMC12223361

[3] R. A. Leon‐Ferre and M. P. Goetz , “Advances in Systemic Therapies for Triple Negative Breast Cancer,” Bmj 381 (2023): 071674, 10.1136/bmj-2022-071674. 37253507

[4] N. U. Lin , A. Vanderplas , M. E. Hughes , et al., “Clinicopathologic Features, Patterns of Recurrence, and Survival Among Women With Triple‐Negative Breast Cancer in the National Comprehensive Cancer Network,” Cancer 118 (2012): 5463–5472, 10.1002/cncr.27581. 22544643 PMC3611659

[5] Y. Liu , Y. Hu , J. Xue , et al., “Advances in Immunotherapy for Triple‐Negative Breast Cancer,” Molecular Cancer 22 (2023): 145, 10.1186/s12943-023-01850-7. 37660039 PMC10474743

[6] X. Jiang , J. Wang , L. Lin , et al., “Macrophages Promote Pre‐Metastatic Niche Formation of Breast Cancer Through Aryl Hydrocarbon Receptor Activity,” Signal Transduction and Targeted Therapy 9 (2024): 352, 10.1038/s41392-024-02042-5. 39690159 PMC11652640

[7] M. Yi , T. Li , M. Niu , et al., “Exploiting Innate Immunity for Cancer Immunotherapy,” Molecular Cancer 22 (2023): 187, 10.1186/s12943-023-01885-w. 38008741 PMC10680233

[8] Y. Zhou , Y. Wei , X. Tian , and X. Wei , “Cancer Vaccines: Current Status and Future Directions,” Journal of Hematology & Oncology 18 (2025): 18, 10.1186/s13045-025-01670-w. 39962549 PMC11834487

[9] Y. Zhang and Z. Zhang , “The History and Advances in Cancer Immunotherapy: Understanding the Characteristics of Tumor‐Infiltrating Immune Cells and Their Therapeutic Implications,” Cellular & Molecular Immunology 17 (2020): 807–821, 10.1038/s41423-020-0488-6. 32612154 PMC7395159

[10] H. du Bois , T. A. Heim , and A. W. Lund , “Tumor‐Draining Lymph Nodes: at the Crossroads of Metastasis and Immunity,” Science Immunology 6 (2021): abg3551, 10.1126/sciimmunol.abg3551. PMC8628268 34516744

[11] B. M. Kahn , R. W. S. Ng , I. K. Kim , et al., “Intrinsic Properties of the Lymph Node Render it Immunologically Susceptible to Metastasis,” Cancer Discovery 15 (2025): 1949–1968. 40265527 10.1158/2159-8290.CD-24-1847 PMC12409284

[12] Q. Huang , X. Wu , Z. Wang , et al., “The Primordial Differentiation of Tumor‐Specific Memory CD^8+^ T Cells as Bona Fide Responders to PD‐1/PD‐L1 Blockade in Draining Lymph Nodes,” Cell 185 (2022): 4049–4066. 36208623 10.1016/j.cell.2022.09.020

[13] I. Delclaux , K. S. Ventre , D. Jones , and A. W. Lund , “The Tumor‐Draining Lymph Node as a Reservoir for Systemic Immune Surveillance,” Trends in Cancer 10 (2024): 28–37, 10.1016/j.trecan.2023.09.006. 37863720 PMC10843049

[14] D. Olmeda , D. Cerezo‐Wallis , E. Castellano‐Sanz , S. García‐Silva , H. Peinado , and M. S. Soengas , “Physiological Models for In Vivo Imaging and Targeting the Lymphatic System: Nanoparticles and Extracellular Vesicles,” Advanced Drug Delivery Reviews 175 (2021): 113833, 10.1016/j.addr.2021.113833. 34147531

[15] Y. Song , L. Bugada , R. Li , et al., “Albumin Nanoparticle Containing a PI3Kγ Inhibitor and Paclitaxel in Combination With α‐PD1 Induces Tumor Remission of Breast Cancer in Mice,” Science Translational Medicine 14 (2022): abl3649, 10.1126/scitranslmed.abl3649. PMC9589917 35507675

[16] T. Karakousi , T. Mudianto , and A. W. Lund , “Lymphatic Vessels in the Age of Cancer Immunotherapy,” Nature Reviews Cancer 24 (2024): 363–381, 10.1038/s41568-024-00681-y. 38605228 PMC12312704

[17] H. Zhou , P. J. Lei , and T. P. Padera , “Progression of Metastasis Through Lymphatic System,” Cells 10 (2021): 627. 33808959 10.3390/cells10030627 PMC7999434

[18] L. Shang , Y. Zhong , Y. Yao , et al., “Subverted Macrophages in the Triple‐Negative Breast Cancer Ecosystem,” Biomedicine & Pharmacotherapy 166 (2023): 115414, 10.1016/j.biopha.2023.115414. 37660651

[19] D. G. DeNardo and B. Ruffell , “Macrophages as Regulators of Tumour Immunity and Immunotherapy,” Nature Reviews Immunology 19 (2019): 369–382, 10.1038/s41577-019-0127-6. PMC7339861 30718830

[20] S. Lamorte , R. Quevedo , R. Jin , et al., “Lymph Node Macrophages Drive Immune Tolerance and Resistance to Cancer Therapy by Induction of the Immune‐Regulatory Cytokine IL‐33,” Cancer Cell 43 (2025): 955–969, 10.1016/j.ccell.2025.02.017. 40054466 PMC12074877

[21] H. W. Wang and J. A. Joyce , “Alternative Activation of Tumor‐Associated Macrophages by IL‐4,” Cell Cycle 9 (2010): 4824–4835, 10.4161/cc.9.24.14322. 21150330 PMC3047808

[22] I. Vitale , G. Manic , L. M. Coussens , G. Kroemer , and L. Galluzzi , “Macrophages and Metabolism in the Tumor Microenvironment,” Cell Metabolism 30 (2019): 36–50, 10.1016/j.cmet.2019.06.001. 31269428

[23] P. Ordentlich , “Clinical Evaluation of Colony‐Stimulating Factor 1 Receptor Inhibitors,” Seminars in Immunology 54 (2021): 101514, 10.1016/j.smim.2021.101514. 34776301

[24] S. M. Pyonteck , L. Akkari , A. J. Schuhmacher , et al., “CSF‐1R Inhibition Alters Macrophage Polarization and Blocks Glioma Progression,” Nature Medicine 19 (2013): 1264–1272, 10.1038/nm.3337. PMC3840724 24056773

[25] C. Pfirschke , R. Zilionis , C. Engblom , et al., “Macrophage‐Targeted Therapy Unlocks Antitumoral Cross‐Talk Between IFNγ‐Secreting Lymphocytes and IL12‐Producing Dendritic Cells,” Cancer Immunology Research 10 (2022): 40–55, 10.1158/2326-6066.CIR-21-0326. 34795032 PMC10132467

[26] Y. N. Lamb , “Pexidartinib: First Approval,” Drugs 79 (2019): 1805–1812, 10.1007/s40265-019-01210-0. 31602563 PMC7044138

[27] R. Roskoski Jr. , “Properties of FDA‐Approved Small Molecule Protein Kinase Inhibitors: A 2021 Update,” Pharmacological Research 165 (2021): 105463, 10.1016/j.phrs.2021.105463. 33513356

[28] N. Zhu , S. Chen , Y. Jin , et al., “Enhancing Glioblastoma Immunotherapy With Integrated Chimeric Antigen Receptor T Cells Through the Re‐Education of Tumor‐Associated Microglia and Macrophages,” ACS Nano 18 (2024): 11165–11182, 10.1021/acsnano.4c00050. 38626338

[29] Z. Li , Y. Ding , J. Liu , et al., “Depletion of Tumor Associated Macrophages Enhances Local And Systemic Platelet‐Mediated Anti‐PD‐1 Delivery for Post‐Surgery Tumor Recurrence Treatment,” Nature Communications 13 (2022): 1845, 10.1038/s41467-022-29388-0. PMC8987059 35387972

[30] N. Butowski , H. Colman , J. F. De Groot , et al., “Orally Administered Colony Stimulating Factor 1 Receptor Inhibitor PLX3397 in Recurrent Glioblastoma: An Ivy Foundation Early Phase Clinical Trials Consortium Phase II Study,” Neuro‐Oncology 18 (2016): 557–564, 10.1093/neuonc/nov245. 26449250 PMC4799682

[31] J. MacMicking , Q. W. Xie , and C. Nathan , “Nitric Oxide And Macrophage Function,” Annual Review of Immunology 15 (1997): 323–350, 10.1146/annurev.immunol.15.1.323. 9143691

[32] P. J. Murray and T. A. Wynn , “Protective and Pathogenic Functions of Macrophage Subsets,” Nature Reviews Immunology 11 (2011): 723–737, 10.1038/nri3073. PMC3422549 21997792

[33] C. Lu , S. Liao , B. Chen , et al., “Responsive Probes for In Vivo Magnetic Resonance Imaging of Nitric Oxide,” Nature Materials 24 (2025): 133–142, 10.1038/s41563-024-02054-0. 39587281

[34] M. Feng , W. Jiang , B. Y. S. Kim , C. C. Zhang , Y. X. Fu , and I. L. Weissman , “Phagocytosis Checkpoints as New Targets for Cancer Immunotherapy,” Nature Reviews Cancer 19 (2019): 568–586, 10.1038/s41568-019-0183-z. 31462760 PMC7002027

[35] S. Jaiswal , C. H. Jamieson , W. W. Pang , et al., “CD47 Is Upregulated on Circulating Hematopoietic Stem Cells and Leukemia Cells to Avoid Phagocytosis,” Cell 138 (2009): 271–285, 10.1016/j.cell.2009.05.046. 19632178 PMC2775564

[36] R. Majeti , M. P. Chao , A. A. Alizadeh , et al., “CD47 Is an Adverse Prognostic Factor and Therapeutic Antibody Target on Human Acute Myeloid Leukemia Stem Cells,” Cell 138 (2009): 286–299, 10.1016/j.cell.2009.05.045. 19632179 PMC2726837

[37] N. Gül and M. van Egmond , “Antibody‐Dependent Phagocytosis of Tumor Cells by Macrophages: A Potent Effector Mechanism of Monoclonal Antibody Therapy of Cancer,” Cancer Research 75 (2015): 5008–5013. 26573795 10.1158/0008-5472.CAN-15-1330

[38] M. Song , J. Tian , L. Wang , et al., “Efficient Delivery of Lomitapide Using Hybrid Membrane‐Coated Tetrahedral DNA Nanostructures for Glioblastoma Therapy,” Advanced Materials 36 (2024): 2311760. 10.1002/adma.202311760 38569065

[39] L. Hou , X. Gong , J. Yang , H. Zhang , W. Yang , and X. Chen , “Hybrid‐Membrane‐Decorated Prussian Blue for Effective Cancer Immunotherapy via Tumor‐Associated Macrophages Polarization and Hypoxia Relief,” Advanced Materials 34 (2022): 2200389, 10.1002/adma.202200389. 35103352

[40] B. Li , W. Wang , L. Zhao , et al., “Aggregation‐Induced Emission‐Based Macrophage‐Like Nanoparticles for Targeted Photothermal Therapy and Virus Transmission Blockage in Monkeypox,” Advanced Materials 36 (2024): 2305378. 10.1002/adma.202305378 37931029

[41] G. Ma , S. Du , X. Li , et al., “pH‐Responsive Neutrophil Membrane Camouflage Ga–Mn Bimetallic Nanodecoy Triggers Apoptosis‐Immunity‐Metastasis Suppression for Tumor Therapy,” Biomaterials 327 (2026): 123794, 10.1016/j.biomaterials.2025.123794. 41151369

[42] S. Du , G. Ma , X. Li , et al., “Targeting ROS‐Metabolism Dual Pathways to Trigger PANoptosis by Cobalt–Vanadium Oxides Biomimetic Nanocakes for Tumor Immunotherapy,” Biomaterials 326 (2026): 123726, 10.1016/j.biomaterials.2025.123726. 40987137

[43] K. H. Min , J. H. Kim , S. M. Bae , et al., “Tumoral Acidic pH‐Responsive MPEG‐poly(β‐amino ester) Polymeric Micelles for Cancer Targeting Therapy,” Journal of Controlled Release 144 (2010): 259–266, 10.1016/j.jconrel.2010.02.024. 20188131

[44] X. Xu , Z. Zhang , J. Du , et al., “Recruiting T‐Cells Toward the Brain for Enhanced Glioblastoma Immunotherapeutic Efficacy by Co‐Delivery of Cytokines and Immune Checkpoint Antibodies With Macrophage‐Membrane‐Camouflaged Nanovesicles,” Advanced Materials 35 (2023): 2209785. 10.1002/adma.202209785 37101060

[45] Z. Zhang , X. Xu , J. Du , et al., “Redox‐Responsive Polymer Micelles Co‐Encapsulating Immune Checkpoint Inhibitors and Chemotherapeutic Agents for Glioblastoma Therapy,” Nature Communications 15 (2024): 1118, 10.1038/s41467-024-44963-3. PMC10847518 38320994

[46] K. Poudel , A. Banstola , M. Gautam , et al., “Macrophage‐Membrane‐Camouflaged Disintegrable and Excretable Nanoconstruct for Deep Tumor Penetration,” ACS Applied Materials & Interfaces 12 (2020): 56767–56781, 10.1021/acsami.0c17235. 33289550

[47] D. Nie , Z. Dai , J. Li , et al., “Cancer‐Cell‐Membrane‐Coated Nanoparticles With a Yolk–Shell Structure Augment Cancer Chemotherapy,” Nano Letters 20 (2020): 936–946, 10.1021/acs.nanolett.9b03817. 31671946

[48] C. Kong , C. Luo , F. Xue , et al., “Ultrasound‐Triggered Charge‐Reversal Nanoparticles via Golgi‐Dependent Iterative Transcytosis for Enhanced Deep Tumor Penetration,” ACS Nano 20 (2026): 4741–4757, 10.1021/acsnano.5c14557. 41619197

[49] C. Wang , B. Wu , Y. Wu , X. Song , S. Zhang , and Z. Liu , “Camouflaging Nanoparticles With Brain Metastatic Tumor Cell Membranes: A New Strategy to Traverse Blood–Brain Barrier for Imaging and Therapy of Brain Tumors,” Advanced Functional Materials 30 (2020): 1909369, 10.1002/adfm.201909369.

[50] Y. Zhao , T. Liu , Y. Wang , et al., “Cancer Cell Membrane‐Coated Homodimer Prodrug Nanoassemblies to Simultaneously Deliver Prodrugs and Immune Adjuvants for Combined Chemo‐Immunotherapy,” ACS Nano 19 (2025): 23276–23293, 10.1021/acsnano.5c06202. 40518859

[51] Y. Wang , J. Yu , Z. Luo , et al., “Engineering Endogenous Tumor‐Associated Macrophage‐Targeted Biomimetic Nano‐RBC to Reprogram Tumor Immunosuppressive Microenvironment for Enhanced Chemo‐Immunotherapy,” Advanced Materials 33 (2021): 2103497. 10.1002/adma.202103497 34387375

[52] Y. Qian , S. Qiao , Y. Dai , et al., “Molecular‐Targeted Immunotherapeutic Strategy for Melanoma via Dual‐Targeting Nanoparticles Delivering Small Interfering RNA to Tumor‐Associated Macrophages,” ACS Nano 11 (2017): 9536–9549, 10.1021/acsnano.7b05465. 28858473

