# Supplementary material for: Lighting up Macrophage Reprogramming Assists Immunosuppressive Niche Modulation in Primary Tumors and Tumor‐Draining Lymph Nodes of Breast Cancer
Source: Adv Sci (Weinh). 2026 May 6;13(43):e75593. doi: 10.1002/advs.75593 (PMC13335797; doi:10.1002/advs.75593)
Supplement: Supplementary file 1 — Supporting File: advs75593‐sup‐0001‐SuppMat.pdf. [file ADVS-13-e75593-s001.pdf]

Supplemental information

Lighting up Macrophage Reprogramming Assists Immunosuppressive Niche Modulation in Primary Tumors and Tumor-Draining Lymph Nodes of Breast Cancer

*Yingbo Li, Di Chang, Jie Yang, Ying Bai, Min Chen, Daoshuang Li, Zuoyu Xu, Jiaxin Yuan, Xiaoxuan Xu, Zhiqi Zhang, Yize Li, Zixin Chen, Jinbing Xie, Zebin Xiao, and Shenghong Ju**

Dr. Y. Li, Dr. J. Yang, Dr. J. Yuan, Dr. X. Xu, Dr. Z. Zhang, Dr. Y. Li, Dr. Z. Chen, Prof. J. Xie, Dr. Z. Xiao, Prof. S. Ju
Department of Radiology, Zhongda Hospital, Medical School of Southeast University, Nurturing Center of Jiangsu Province for State Laboratory of AI Imaging & Interventional Radiology, 87 DingJiaQiao Road, Nanjing 210009, China.
E-mail: jsh@seu.edu.cn

Dr. D. Chang,
Department of Radiology, Zhongda Hospital, Medical School of Southeast University, Nurturing Center of Jiangsu Province for State Laboratory of AI Imaging & Interventional Radiology, Collaborative Innovation Center of Radiation Medicine of Jiangsu Higher Education Institutions, 87 DingJiaQiao Road, Nanjing 210009, China.

Dr. Y. Bai
Department of Pharmacology, Jiangsu Provincial Key Laboratory of Critical Care Medicine, School of Medicine, Southeast University, Nanjing 210009, China.

Dr. M. Chen
Department of Radiology, The First Affiliated Hospital of Kangda College of Nanjing Medical University/The First People's Hospital of Lianyungang, Lianyungang, Jiangsu 222000, China

Dr. D. Li
Department of Ultrasound, the Fourth Hospital of Harbin Medical University, Harbin 150001, China.

Dr. Z. Xu
Department of Nuclear Medicine, the Fourth Hospital of Harbin Medical University, Harbin 150023, China.

Materials and methods

Reagents and materials

The CD47 antibody (aCD47) was purchased from BioXcell. DSPE-PEG-Mannose were synthesized by Xi'an Ruixi. (Xi'an, China). The mPEG-poly(β-amino ester) (mPEG-PAE) block copolymer was synthesized by Xi'an Ruixi Biological Technology Co., Ltd. The mPEG block has a molecular weight of 5000 Da, and the PAE block has a molecular weight of 10000 Da, with a total molecular weight of approximately 15000 Da. IL-4 was purchased from PeproTech (USA). IL-10, IL-12, TNF-α and TGF-β ELISA kits were obtained from Elabscience (Wuhan, China). For Western blotting and IF analyses, the following antibodies were used: CD206 (GB115273, Servicebio), CD86 (ab317266, Abcam), ARG1 (93668T, CST), iNOS (ab205529, Abcam), GAPDH (2118S, CST), and HRP-conjugated goat anti-rabbit IgG (RS0002, ImmunoWay). The antibodies used for flow cytometric analyses were as follows: anti-mouse CD11b-PerCP-Cy5.5 (M1/70, 45-0112-82, eBioscience), anti-mouse F4/80-FITC (BM8, 11-4801-82, eBioscience), anti-mouse CD206-PE (MR6F3, 12-2061-82, eBioscience), anti-mouse CD86-APC (GL1, 17-0862-82, eBioscience), anti-human CD206-PE (19.2, 12-2069-42, eBioscience), anti-human CD86-APC (IT2.2, 17-0869-42, eBioscience), Cell Counting Kit-8 (CCK-8) was purchased from DojinDo Molecular Technology (Shanghai, China). Alex647 was acquired from ThermoFisher (MA, USA). Dulbecco's modified Eagle's medium (DMEM), foetal bovine serum (FBS), 1% penicillin and streptomycin, 0.25% trypsin-ethylenediaminetetraacetic acid (EDTA) were purchased from Thermo Fisher Scientific (Thermo Fisher Scientific, Inc., USA). Analytical grades of all other chemicals were obtained from Sigma-Aldrich (Sigma-Aldrich, USA). Schematic illustrations were created with BioRender (BioRender, CA).

TCGA data analysis

The breast cancer patient data were obtained from TCGA-BRCA cohort. The immune landscape of the TNBC microenvironment and immune cell abundance in the TCGA BRCA cohort were analyzed to characterize its features and evaluate the prognostic significance of

TAMs in TNBC. A heatmap was generated to identify patterns and differences in the distribution of immune cell populations across samples.

Synthesis of PA NPs

The macrophage membrane (MM) was isolated from RAW 264.7 cells.[1] In brief, raw 264.7 cells were harvested and resuspended in prechilled Tris-magnesium buffer (TM buffer, pH 7.4) and extruded 11 times through the microextruder (Avanti Polar Lipids, USA) without polycarbonate membrane to destroy the cells. The homogenate of cells was extracted by sucrose gradient centrifugation to collect cell membrane. The total protein content in the purified MM was analyzed by BCA protein assay (KGP902, Nanjing keygen Biotech Co. Ltd., China). For MM modification, DSPE-PEG-Man was incubated with MM at a weight ratio of 1:1.5 to obtain modified MM (MM-Man).

IR NO was synthesized according previous methods.[2] Briefly, IR 1061 and DABT (3,4-diaminobenzenethiol) were dissolved in DCM, then 200 μL triethylamine was added. The mixture was stirred for 12 h at room temperature, followed by the removement of solvent under reduced pressure. The crude product was purified via silica gel flash column chromatography to obtain IR NO as a black powder.

To fabricate drug-loaded micelles, mPEG-poly (β-amino ester) block copolymer (mPEG-PAE) was dissolved in chloroform, followed by adding PLX3397 into the solution. The suspension was then stirred for 60 min at room temperature (RT) to obtain a homogeneous solution. After that, the solvent was evaporated by a rotary evaporator, and a thin film was then obtained. After adding 10 mL of distilled water, the suspension was gently stirred for 2 h and then was sonicated on ice using a probe sonicator for 10 min to get the drugloaded micelles. To further purify the micelles, we filtered out large micelles through dialysis (molecular weight cutoff = 10000, Spectrum). The final products were freeze-dried and stored at −20 °C for further applications.

Next, the mixtures were extruded through a membrane extruder (Avanti Polar Lipids, USA) to prepare PA NPs followed by extraction and purification. During the process of extrusion, sodium cholate (1.5%), cholesterol (0.02%), leupeptin (0.2 μg/ml) and Nacl (500 mM) were added to the reaction system to make them more flexible and minimize the

degradation of membrane proteins. After purified by dialysis, DSPE-PEG-Man was added and the reaction was continued for 2 h to obtain the PA NPs. To quantitatively evaluate the loading capacity of aCD47 in PA NPs, the aCD47 antibody was labeled with Alexa Fluor 647 NHS ester. The labeled aCD47 was then incorporated during the nanoparticle fabrication process to enable encapsulation into PA NPs. After purification to remove free aCD47, the fluorescence intensity of aCD47 in PA NPs and in the supernatant was measured using a fluorescence microplate reader (Thermo Fisher Scientific, USA).

The encapsulation efficiency (EE%) and loading capacity (LC%) of aCD47 were calculated using the following formulas:

$$\text{encapsulation efficiency (\%)} = \frac{amount\ of\ loaded\ drug}{amount\ of\ drug\ added} \times 100\%$$

$$\text{loading efficiency (\%)} = \frac{amount\ of\ loaded\ drug}{amount\ of\ drug\ loaded\ nano\ vesicles} \times 100\%$$

The encapsulation efficiency and loading capacity of PLX3397 were determined by UV-Vis spectrophotometry as described above.

Characterization of PA NPs

The transmission electron microscopy (TEM) images of PA NPs were obtained from a transmission electron microscopy (TEM, HITACHI HT7800, Hitachi High Technologies, Japan). The hydrodynamic diameter was determined by dynamic light scatterings (DLS; 90Plus Particle Size Analyzer; Malvern Instruments Ltd., UK) and the surface charge property was acquired by measuring zeta potentials. The average diameters were measured every day for 14 days in three different solutions (pH 7.4 PBS, Dulbecco's modified Eagle medium (DMEM), and DMEM containing 10% fetal bovine serum (FBS)) to evaluate the stability of the PA NPs. The protein profiles of MM and PA NPs were determined by Coomassie Brilliant Blue staining (Sigma-Aldrich, USA). FITC-labeled DSPE-PEG-Mannose was used to verify the efficiency of the modification. The cell uptake of PA NPs by M2-like macrophages with or without DSPE-PEG-Mannose modified was confirmed by CLSM.

In vitro release of PA NPs

To evaluate the release profile of aCD47 and PLX3397, PA NPs loaded with Alexa Fluor 647-labeled aCD47 were placed into dialysis bags (molecular weight cutoff: 300 kDa, Spectrum, USA) and immersed in 50 mL PBS at different pH conditions (pH 7.4 and 6.5) to simulate physiological and tumor microenvironment conditions. The system was maintained at 37 °C under gentle shaking (100 rpm). At predetermined time intervals, aliquots of the release medium were collected and replaced with an equal volume of fresh pre-warmed PBS. The amount of released aCD47 and PLX3397 were quantified by measuring fluorescence intensity using microplate reader and UV-Vis spectrophotometry respectively.

Cell culture and animals

4T1 cells and EMT6 cells were cultured and maintained in RPMI 1640 culture medium, while Raw264.7 cells, NIH 3T3 cells and HC11 cells were cultured and maintained in DMEM. Both medium were supplemented with 10% fetal bovine serum (FBS) and 1% penicillin-streptomycin. All cells were incubated in an atmosphere at 37°C with 5% CO_2.

All animal studies were conducted according to the ethical guidelines of the Animal Care & Welfare Committee of Southeast University, Jiangsu, China.

Flow cytometry analyses

Mice were euthanized at specific time and tumors or TDLNs (inguinal lymph nodes) or spleen were harvested as previously mentioned.[3,4] Briefly, tissues were shredded and digested at 37°C for 1 h. The cells were then filtered with 70-μm cell strainers. Subsequently Fc receptors were blocked with CD16/CD32 antibody for 10 min on ice, and incubated with fixable viability dye for 20 min at 4 °C. Next, samples were stained with fluorescent dye-labeled antibodies purchased from eBioscience. Antibodies were applied for staining according to the manufacturer's suggestions. Finally, cells were washed and analyzed by flow cytometry on the Attune® NxT flow cytometer (Thermo Fisher Scientific). The data were analyzed and quantified by the FlowJo V10 software (BD Biosciences). The gating strategies were displayed in Supplementary Fig. 36.

Enzyme-linked immunoassay

Enzyme-linked immunoassay (ELISA) kits were used to analyze the levels of murine IL-10, murine IL-12, murine TGF-β, murine TNF-α, following the manufacturer's instructions. For solid tissue samples (e.g., tumors or lymph nodes), all measurements were normalized to the total protein concentration to account for variations in sample size and cellularity. Specifically, after homogenization, tissue lysates were clarified and the total protein concentration of each sample was determined using a bicinchoninic acid (BCA) assay. Cytokine levels in these tissues were then expressed as picograms per milligram of total protein (pg/mg). For liquid samples (e.g., serum or cell culture supernatants), cytokine concentrations were measured directly and expressed as picograms per milliliter (pg/ml) following the kit's standard curve.

Macrophage differentiation

For macrophage differentiation, bone marrow derived macrophages (BMDMs) were cultured in DMEM containing 10% heat-inactivated FBS and recombinant murine macrophage colony-stimulating factor for 7 days. Then, BMDMs were stimulated with murine IL-4 (20 ng/mL) for 2 d for M2-like macrophages activation. In addition, to explore the potential of PA NPs for reprograming M2-like macrophages to M1-like phenotype, BMDMs were treated with prepared formulations for 24 h. Next, associated markers (CD206, IL-10, TGF-β, CD86, IL-12, TNF-α) were analyzed by flow cytometry, immunofluorescence or ELISA.

***In vitro* cytotoxicity of PA NPs**

The *in vitro* cytotoxicity of PA NPs and light triggered cytotoxicity of against 4T1 cells were determined by CCK-8 assay. macrophages, NIH-3T3 or HC11 cells (5×10^3 cells per well) were seeded into a 96-well plate and incubated for 24 h. After washing with PBS for 3 times, the cells were incubated with 100 uL culture medium with different concentrations of PA NPs for 24 h. Then the relative viability of 4T1 cells was measured with CCK-8 assay. In short, 10 μL of CCk-8 solution was added to each well with 90 μL medium and incubated for 4 h, and each group was examined in triplicate. The absorbance at 450 nm was analyzed by a microplate reader.

In vivo pharmacokinetics of PA NPs

To investigate pharmacokinetic behavior of PA NPs, free IR825 and IR825 labeled-PA NPs were administrated into healthy SD rats via tail vein (n = 3). Then blood samples were collected at 0.25, 0.5, 1, 2, 4, 8, 12 and 24 h after injection. Plasma was subsequently separated, the concentrations of IR825 were detected using the fluorescence microplate reader. The drug metabolism parameters of the nanoparticles included elimination half-life, area under the curve and clearance rate were calculated using a no-compartment model with the Maspectra v2.10 software.

In vivo and *ex vivo* imaging

In fluorescence imaging experiments, tumor-bearing mice were randomly divided into two groups. The mice were intravenously injected with free IR825 and PA NPs respectively. Then, images were acquired at several time points (1, 2, 4, 8, 12, 24, 48 and 72 h) on the IVIS-Spectrum system (Perkin Elmer, Santa Clara, CA) with an excitation wavelength of 780 nm and an emission wavelength of 845 nm. *In vivo* near infrared (NIR) fluorescence imaging was performed. The *ex vivo* biodistribution of PA NPs in major organs (heart, liver, spleen, lung and kidneys) and tumors was evaluated by *in vivo* fluorescence imaging at 72 h.

For NIR-II imaging, tumor-bearing mice were randomly divided into two groups. The mice were intravenously injected with PLX 3397 free NPs (MM-NO) and PA NPs respectively. Then, NIR-II images were acquired at several time points (0, 6, 12, 24, 48, 72 and 120 h). *In vivo* NIR-II imaging of axillary lymph node and lung metastatic tumors was conducted through a similar method.

Tumor model and *in vivo* biosafety assay

Six- to eight-week-old female BALB/c mice were obtained from Yangzhou University Medical Center (Yangzhou, China). All animal experimental procedures were approved by the Animal Ethics Committee of Southeast University (approval number: 20240321016) and were performed in compliance with the Regulations for the Administration of Affairs Concerning Experimental Animals of China. 4T1 cells or EMT6 cells were injected in the mammary fat pad to induce tumor-bearing mice model. When the tumor volume reached 50~100 mm^3, mice

were randomly divided to different groups. The mice were intravenously injected with different formulations on day 0, 3 and 6. The tumor volume and body weight were monitored and recorded every 2 days. Meanwhile, the survival rate of mice was monitored throughout the experiment. On the 14th day after treatment, six mice of each group were euthanized and the tumors and lymph nodes were resected. Additionally, the tumors were weighed and imaged. The tumor growth inhibition (TGI) was calculated based on the tumor weight. The tumors were collected for further analysis, including H&E staining, TUNEL assay, immunohistochemical staining, immunofluorescence staining and flow cytometry analysis. And lymph nodes were used for flow cytometry analysis.

For biosafety assay, healthy female BALB/c mice were injected with the PA NPs or free aCD47 antibody *via* the tail vein. The blood of posterior orbital venous plexus was collected at continuous time points. H&E staining was performed in the tissues of the major organs of mice (heart, liver, spleen, lung, kidney) to test the *in vivo* biosafety of the PA NPs.

Single-cell RNA-seq library preparation and sequencing

In this study, six fresh specimens—comprising three surgically excised primary TNBC tumors and their corresponding adjacent normal tissues—were collected from three patients to enable single-cell transcriptomic profiling of the tumor microenvironment. Following high quality control, a total of 45,620 single cells were retained, encompassing a diverse range of cell types such as myeloid cells, lymphoid populations (including T and B cells), and so on. Library construction was carried out using the Single Cell 3′ Library and Gel Bead Kit V3.1 in strict accordance with the manufacturer's specifications, and subsequent sequencing was conducted on an Illumina NovaSeq 6000 system, achieving a minimum depth of 100,000 reads per cell under a paired-end 150 bp (PE150) configuration (CapitalBio Technology, Beijing). The single-cell RNA-sequencing data used in this study are publicly available in the Genome Sequence Archive (GSA) under the accession number HRA011711. All experiments using human samples have received approval from the Zhongda Hospital Southeast University Ethics Committee and the Fourth Hospital of Harbin Medical University Ethics Committee according to related ethical guidelines (approval number: 2025ZDSYLL132-P01).

Statistical analysis

Statistical analyses were conducted using t-test, one-way ANOVA. For survival analysis, Kaplan-Meier survival estimation with a log-rank (Mantel-Cox) test was performed. All values were described as the means ± standard deviation (SD), and differences were considered to be statistically significant at * $P < 0.05$, ** $P < 0.01$, *** $P < 0.001$, and **** $P < 0.0001$. All statistical analyses were performed by GraphPad Prism 8.0.

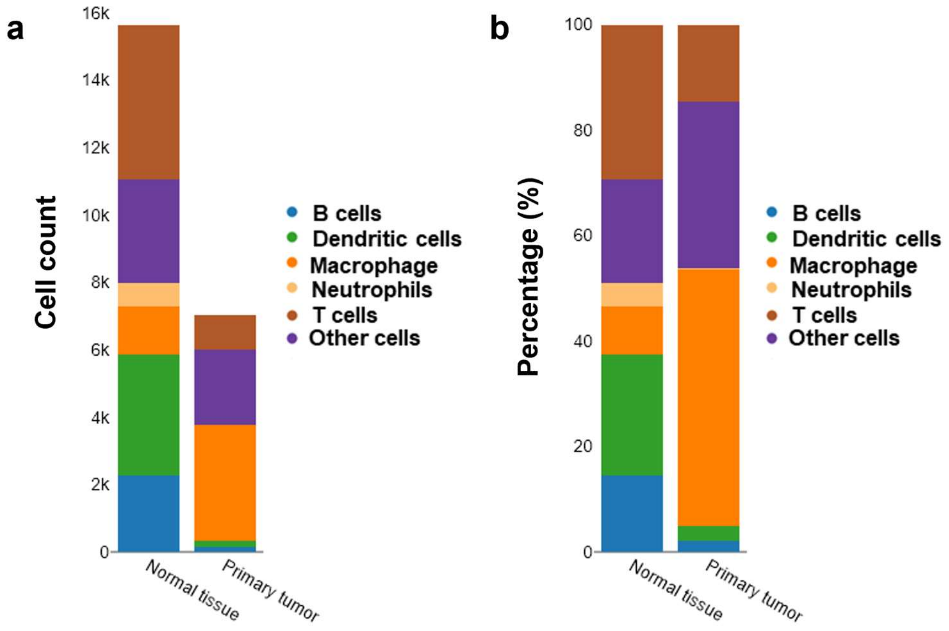

Figure S1. The number and percentage of different cell types in tumor tissues and normal tissues *via* scRNA-seq data.

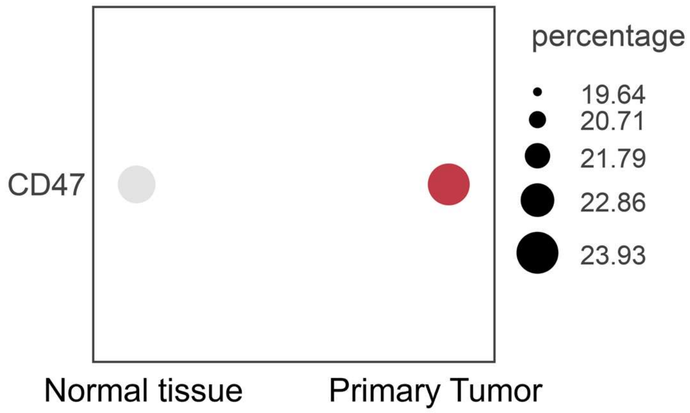

Figure S2. Bubble plot *via* scRNA-seq data depicting the expression level of CD47 in normal and tumor tissues.

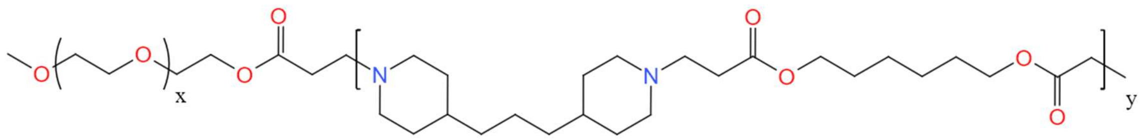

Figure S3. The chemical structure of mPEG-PAE.

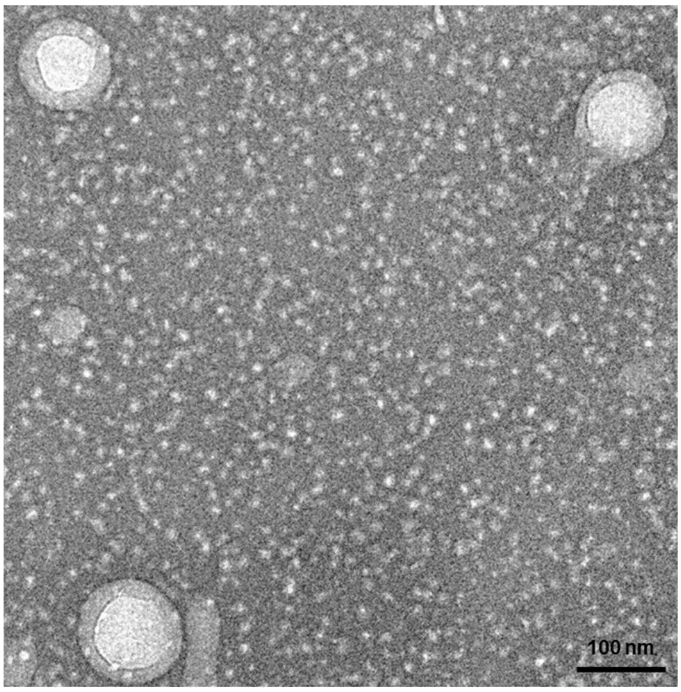

Figure S4. TEM images of PA NPs, the scale bar is 100 nm.

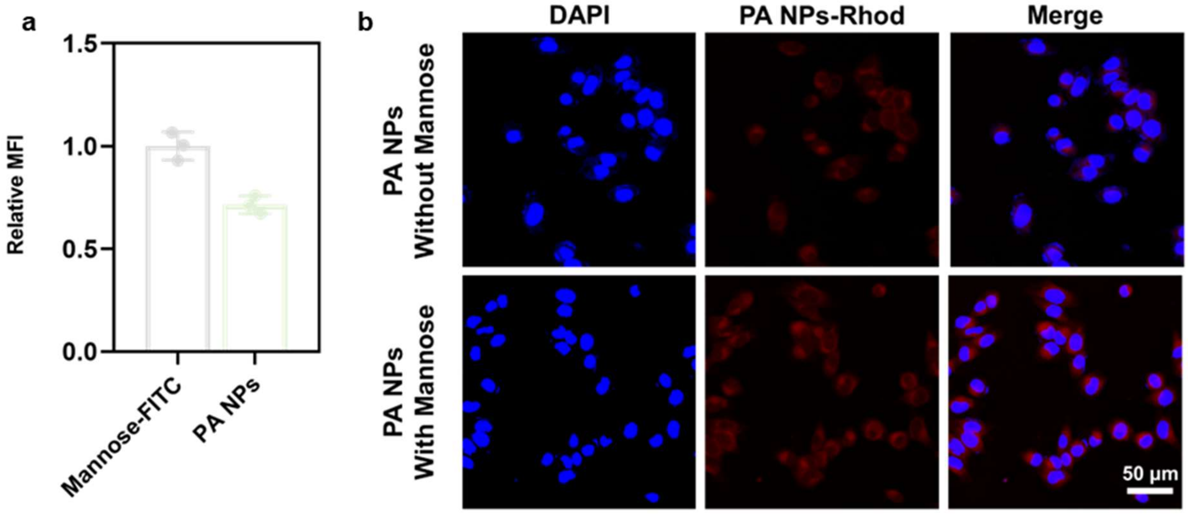

Figure S5. a) The efficiency of DSPE-PEG-Mannose modification. b) Cell uptake of PA NPs with or without DSPE-PEG-Mannose modification by M2-like macrophages. Data are shown as mean ± SD (n = 3).

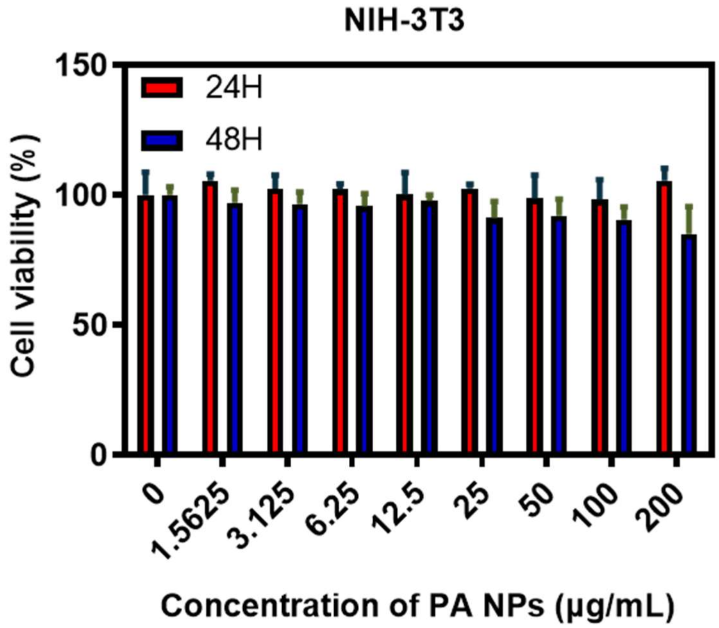

Figure S6. Relative NIH-3T3 cells viability after incubation with different concentration of PA NPs for 24 h and 48 h. Data are shown as mean ± SD (n = 6).

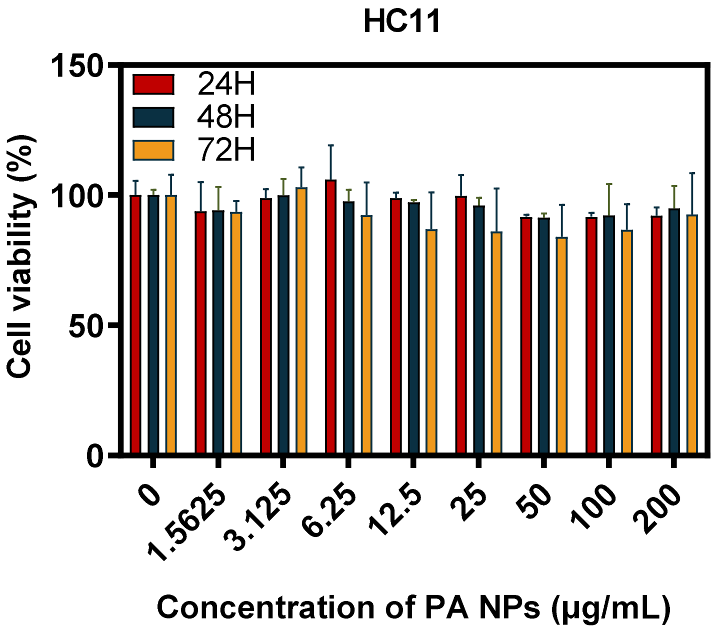

Figure S7. Relative HC11 cells viability after incubation with different concentration of PA NPs for 24 h, 48 h and 72 h. Data are shown as mean ± SD (n = 6).

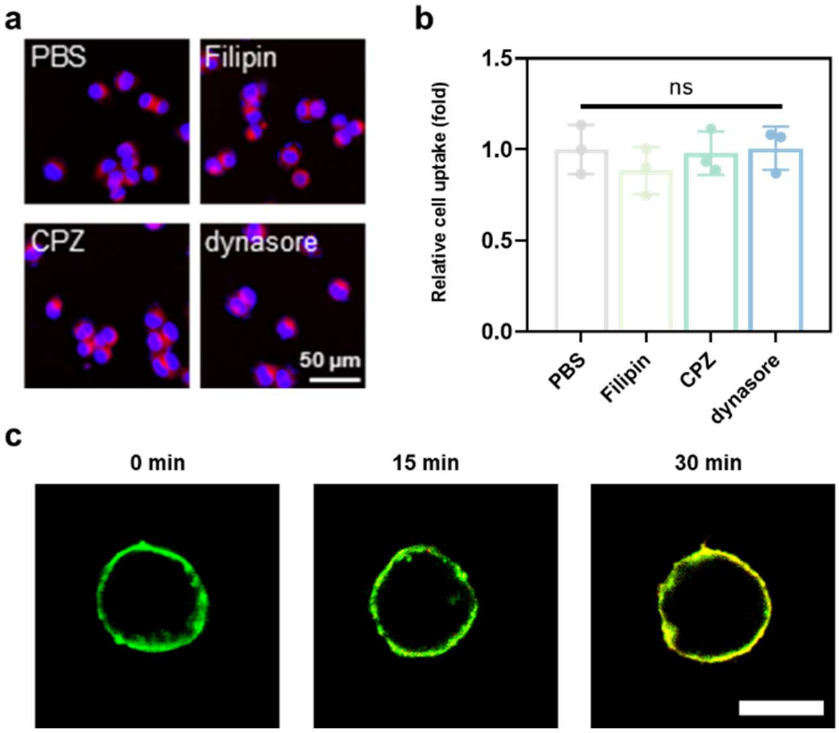

Figure S8. a-b) Cellular uptake of PA NPs after treatment with different endocytosis inhibitors. c) CLSM images indicating the interaction between DiI-labeled NPs and DiO-labeled cell membrane. Scale bar = 10 μm. Statistical significance was determined using one-way ANOVA with Tukey's multiple comparison tests. Data are shown as mean ± SD (ns *P* >0.05).

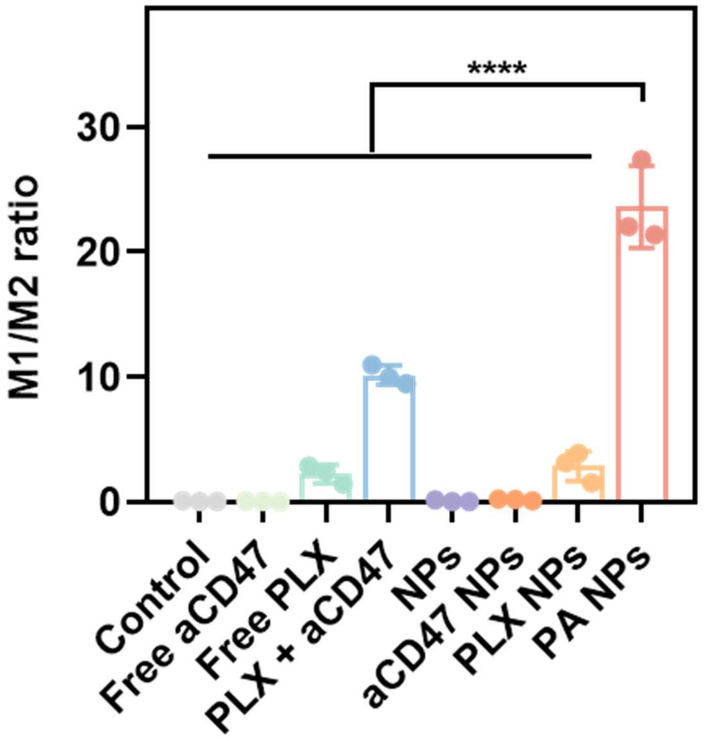

.

Figure S9. Quantification of the expression of CD86 (M1-like phenotype) and CD206 (M2-like phenotype) ratio (M1/M2) in BMDMs after different treatments (n = 3). Statistical significance was determined using one-way ANOVA with Tukey's multiple comparison tests. Data are shown as mean ± SD (**** $P < 0.0001$).

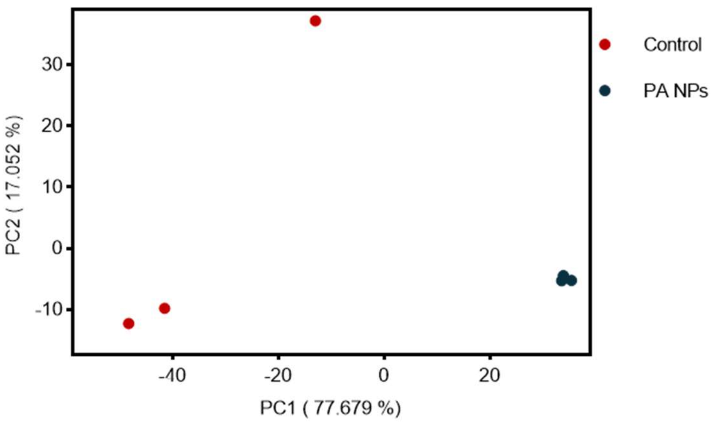

Figure S10. PCA score plot of the expressed genes in BMDM with/without PA NPs treatment (n = 3).

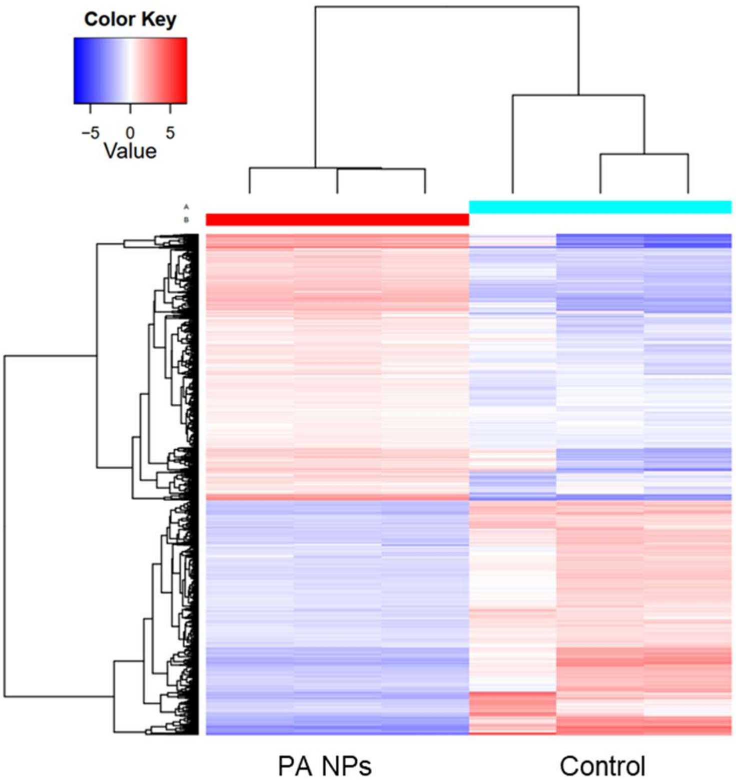

Figure S11. Landscape of DEG distribution in BMDM with/without PA NPs treatment (n = 3).

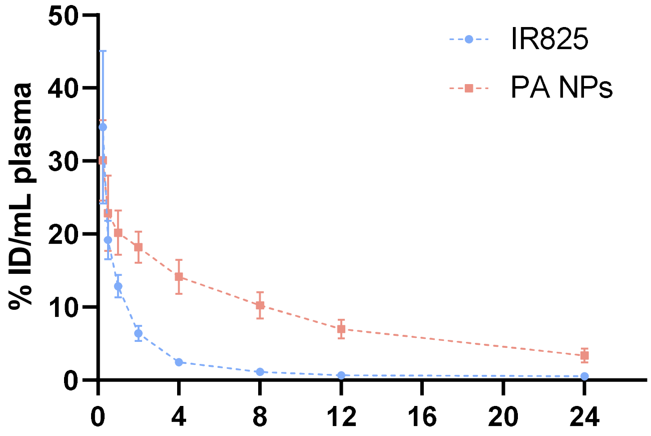

Figure S12. *In vivo* pharmacokinetics of free IR825 and PA NPs (n = 3, 5mg/kg), equivalent to IR825. Data are shown as mean ± SD.

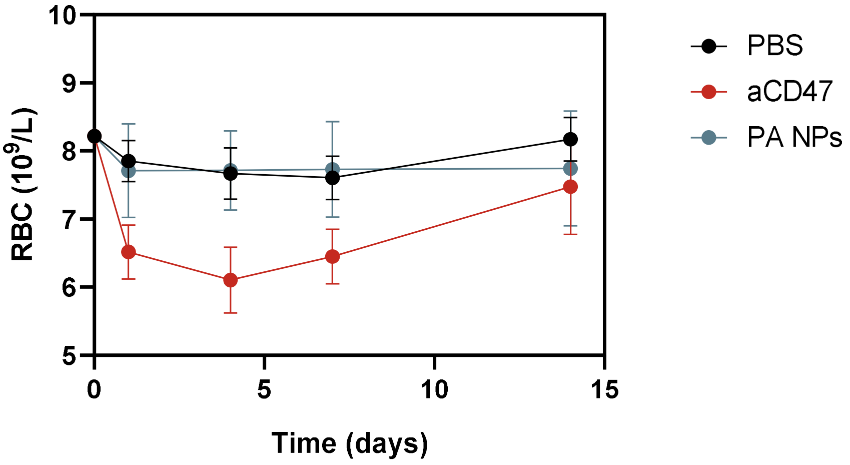

Figure S13. Number of red blood cells (RBC) after different treatment in healthy mice (n = 5). Data are shown as mean ± SD.

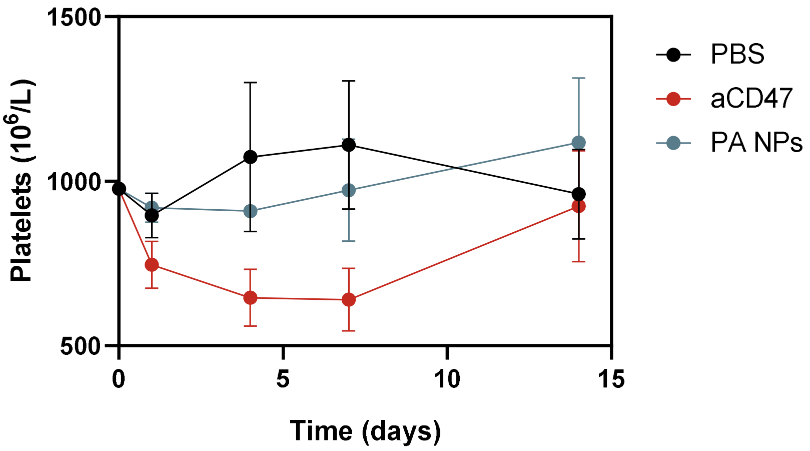

Figure S14. Number of platelets after different treatment in healthy mice (n = 5). Data are shown as mean ± SD.

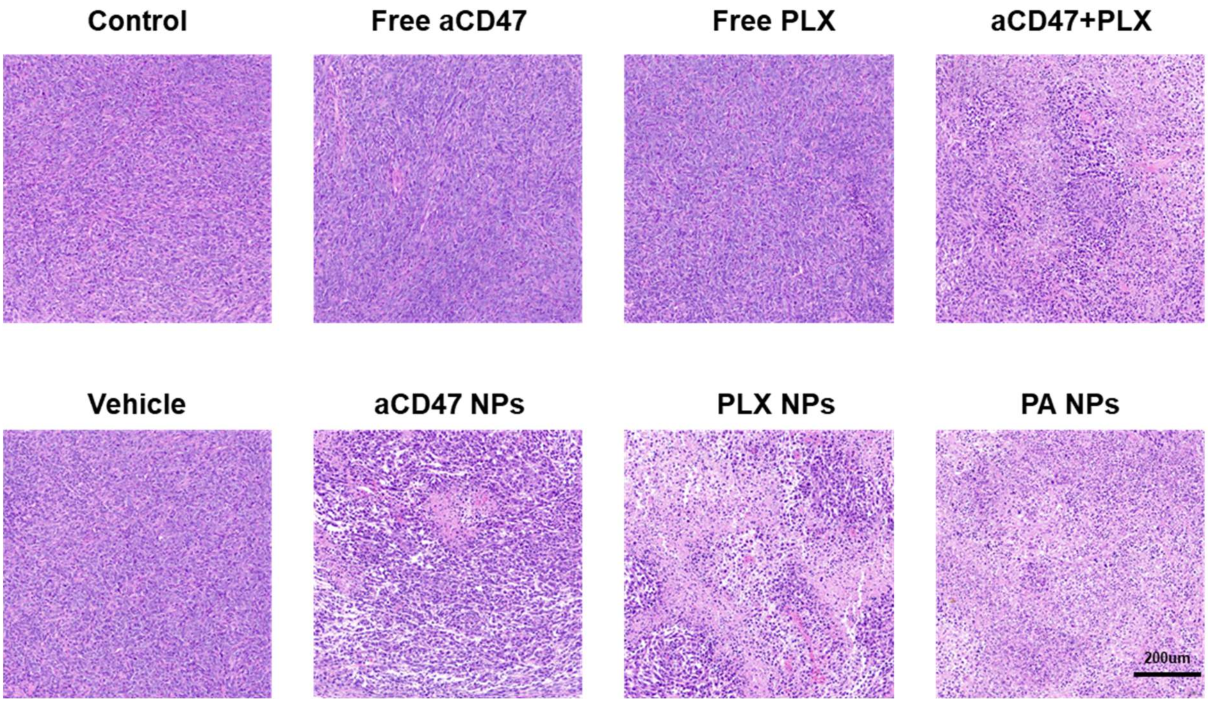

Figure S15. Representative images of H&E staining of tumor sections after different treatments. The scale bar is 200 μm.

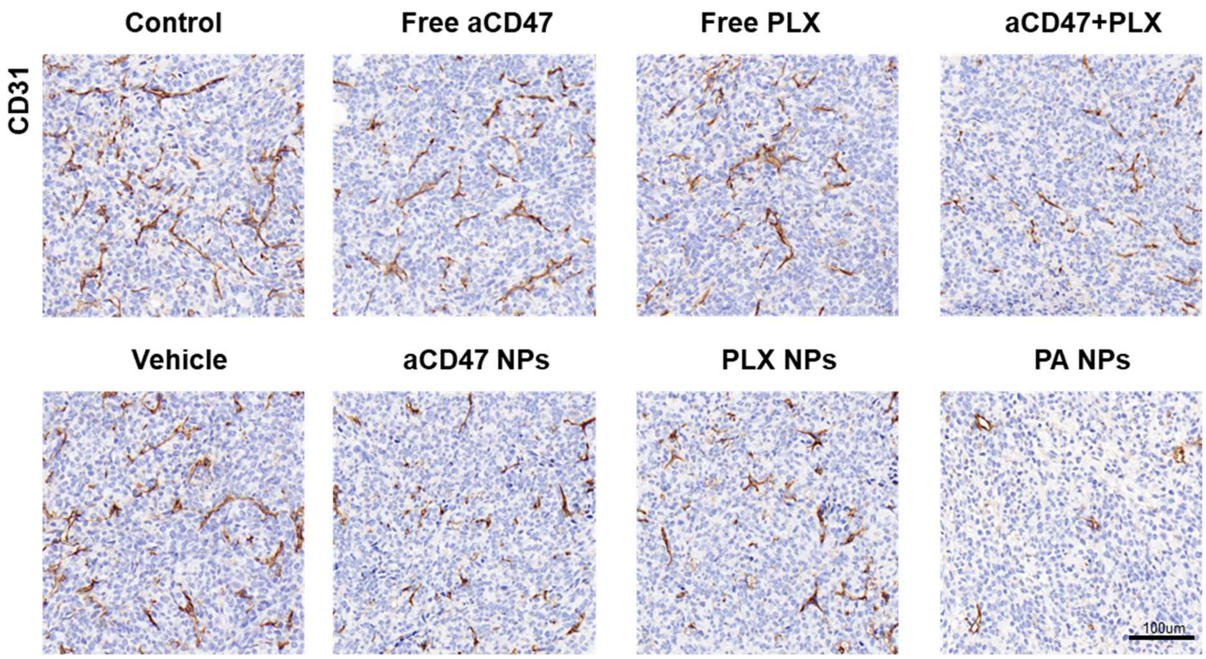

Figure S16. Representative immunohistochemistry images of CD31 staining of tumor sections after different treatments. The scale bar is 100 μm.

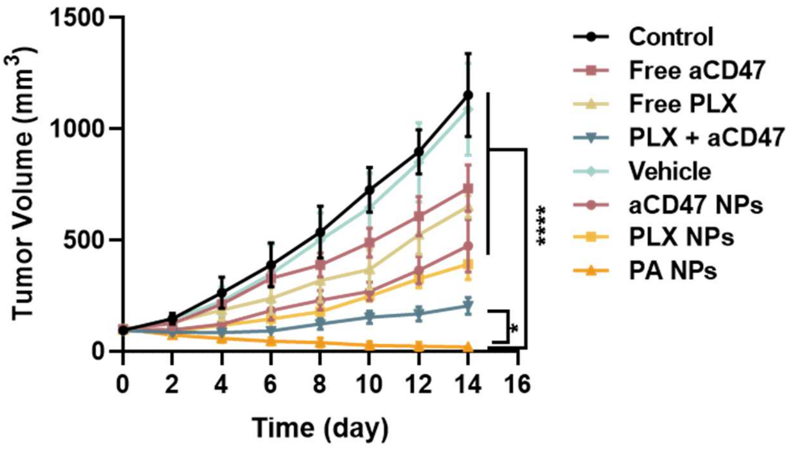

Figure S17. Tumor growth profiles of EMT6-tumor-bearing BALB/c mice after different treatments (n = 6). Statistical significance was determined using one-way ANOVA with Tukey's multiple comparison tests. Data are shown as mean ± SD (* $P < 0.05$, **** $P < 0.0001$).

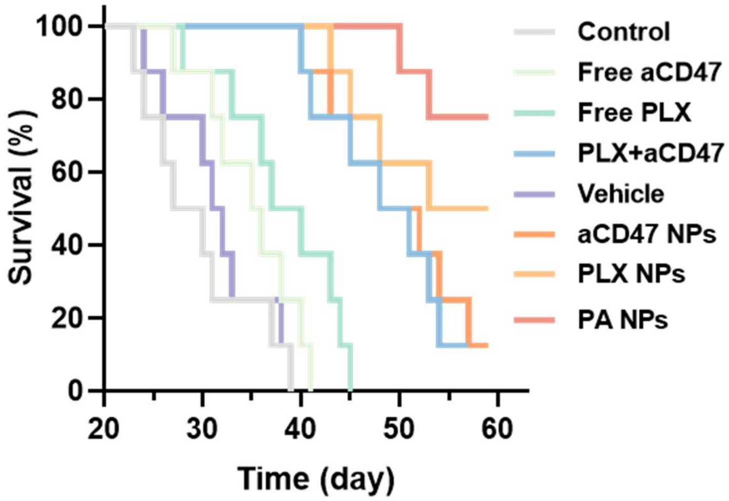

Figure S18. Survival curves of EMT6-tumor-bearing BALB/c mice after different treatments (n = 8).

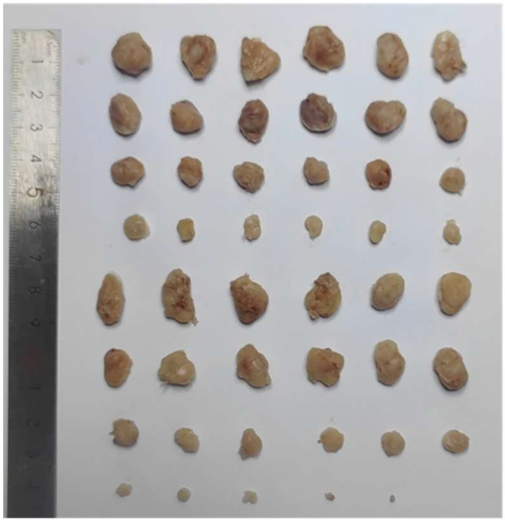

Figure S19. The photograph of tumors excised from EMT6 orthotopic tumor-bearing mice after different treatments on day 14 (n = 6).

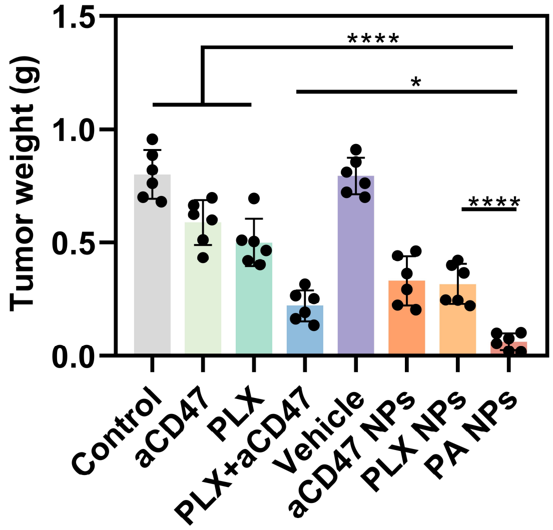

Figure S20. Weight of tumors excised from EMT6 orthotopic tumor-bearing mice on day 14 (n = 6). Statistical significance was determined using one-way ANOVA with Tukey's multiple comparison tests. Data are shown as mean ± SD (* $P < 0.05$, **** $P < 0.0001$).

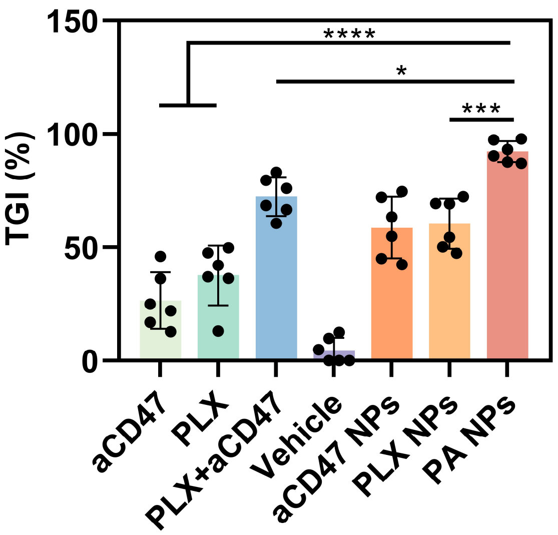

Figure S21. Tumor growth inhibition (TGI) of EMT6 orthotopic tumor-bearing mice after different treatments (n = 6). Statistical significance was determined using one-way ANOVA with Tukey's multiple comparison tests. Data are shown as mean ± SD (* $P < 0.05$, *** $P < 0.001$, **** $P < 0.0001$).

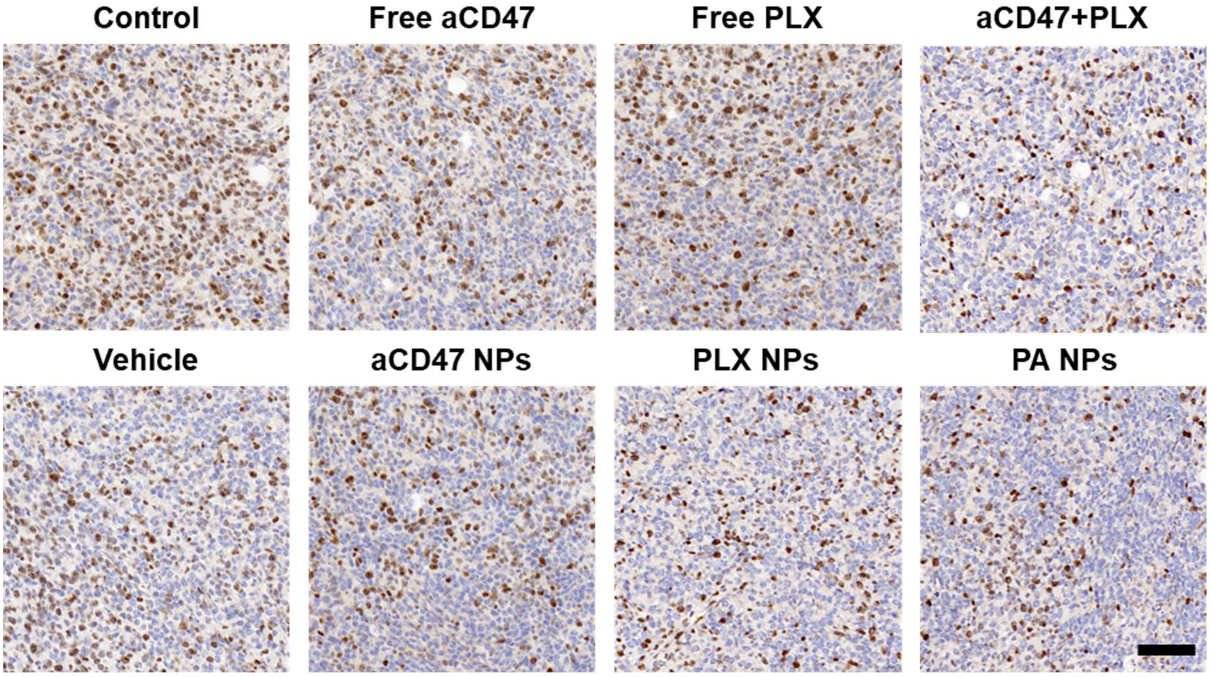

Figure S22. Representative immunohistochemistry images of Ki67 staining of tumor sections after different treatments. The scale bar is 50 μm.

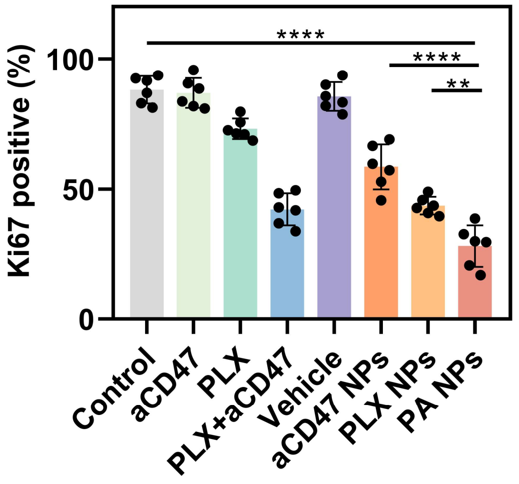

Figure S23. Quantitative analysis of Ki67 staining of tumor sections after different treatments (n = 6). Statistical significance was determined using one-way ANOVA with Tukey's multiple comparison tests. Data are shown as mean ± SD (** $P < 0.01$, **** $P < 0.0001$).

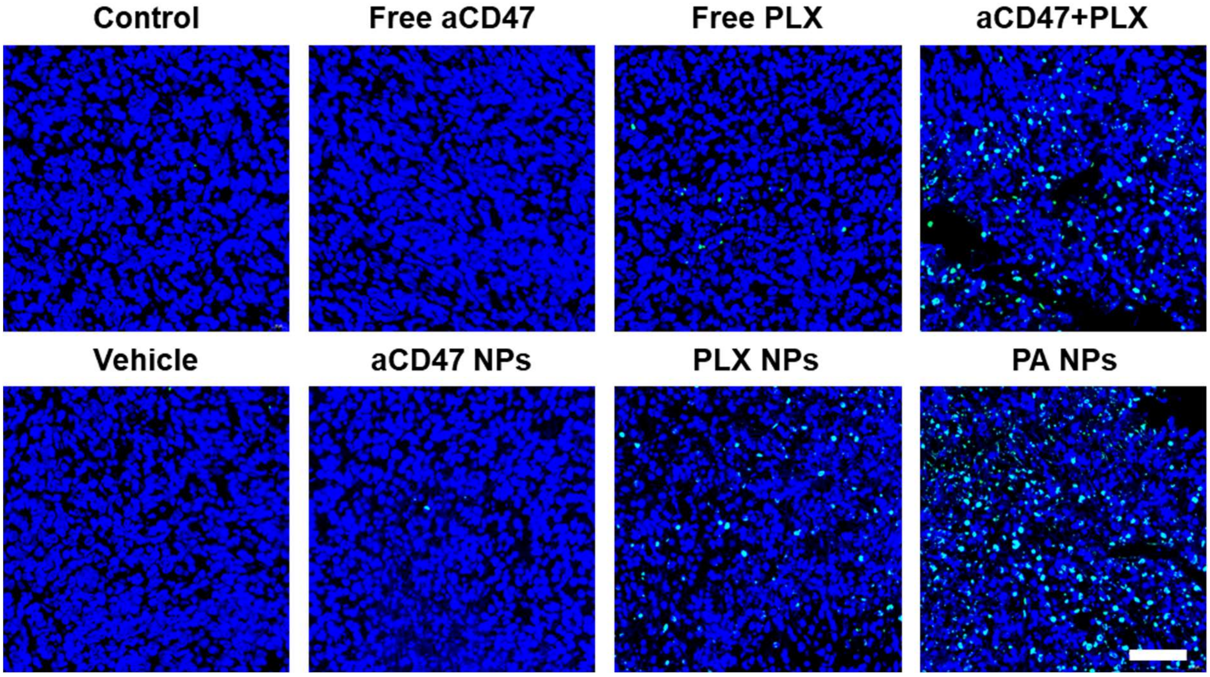

Figure S24. Representative immunohistochemistry images of TUNEL staining of tumor sections after different treatments. The scale bar is 50 μm.

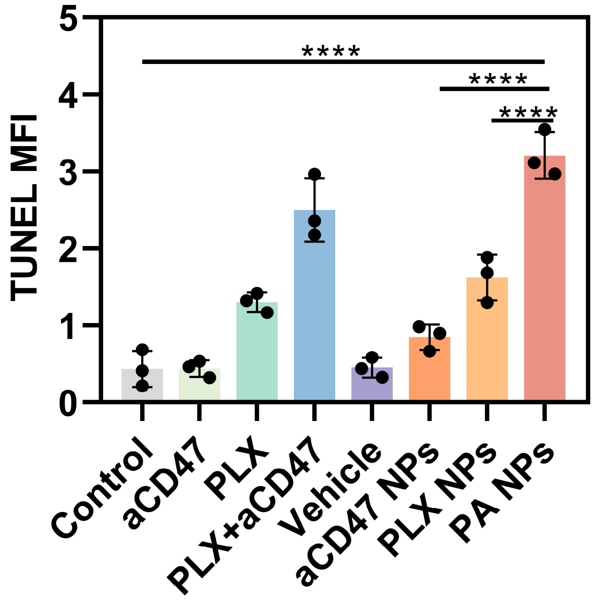

Figure S25. Quantitative analysis of TUNEL staining of tumor sections after different treatments (n = 3). Statistical significance was determined using one-way ANOVA with Tukey's multiple comparison tests. Data are shown as mean ± SD (**** $P < 0.0001$).

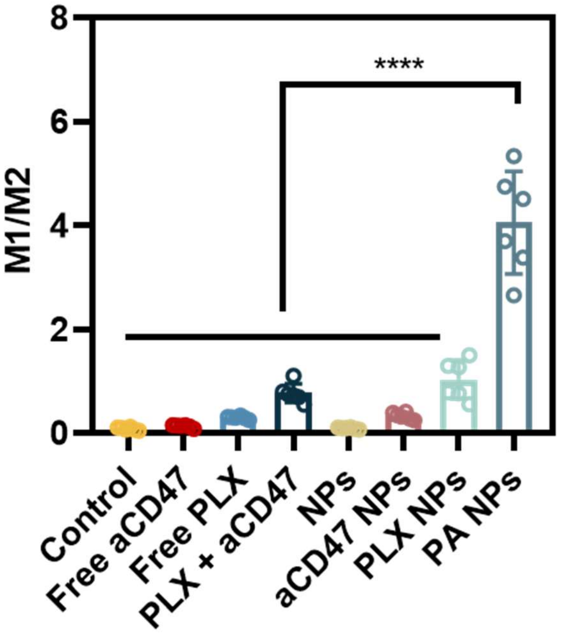

Figure S26. Quantification of flow cytometry of the M1/M2 ratio in 4T1 tumors after different treatments (n = 6). Statistical significance was determined using one-way ANOVA with Tukey's multiple comparison tests. Data are shown as mean ± SD (**** $P < 0.0001$).

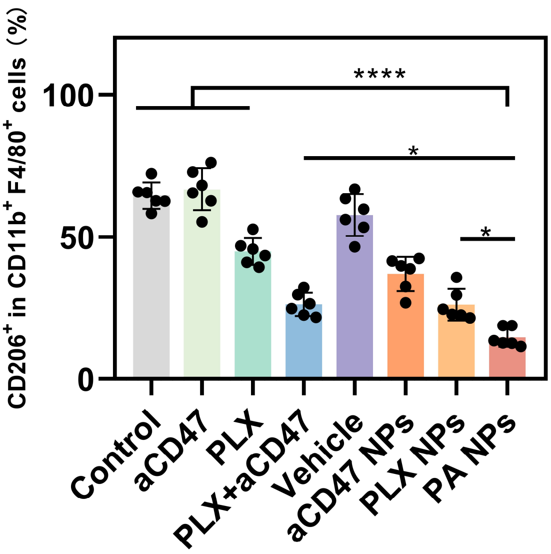

Figure S27. The quantitative analysis of the expression of CD206 in EMT6 tumors after different treatments (n = 6). Statistical significance was determined using one-way ANOVA with Tukey's multiple comparison tests. Data are shown as mean ± SD (*$P < 0.05$, **** $P < 0.0001$).

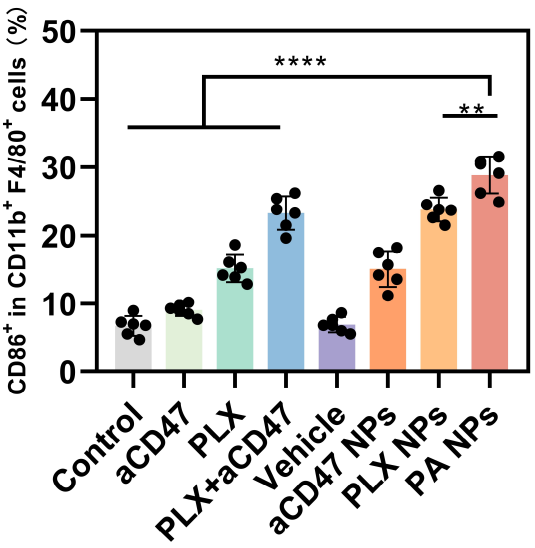

Figure S28. The quantitative analysis of the expression of CD86 in EMT6 tumors after different treatments (n = 6). Statistical significance was determined using one-way ANOVA with Tukey's multiple comparison tests. Data are shown as mean ± SD (**$P < 0.01$, **** $P < 0.0001$).

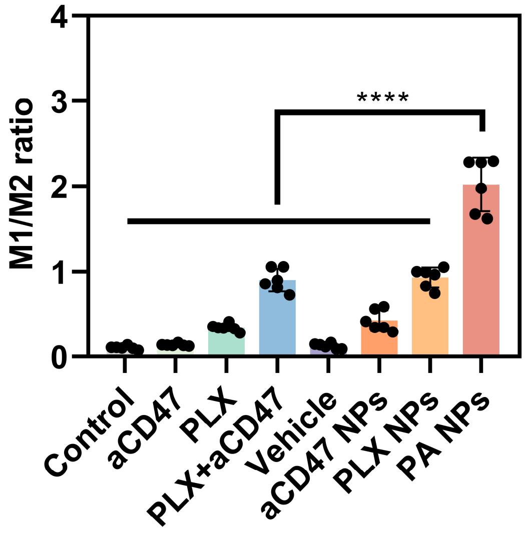

Figure S29. Quantification of flow cytometry of the M1/M2 ratio in EMT6 tumors after different treatments (n = 6). Statistical significance was determined using one-way ANOVA with Tukey's multiple comparison tests. Data are shown as mean ± SD (**** $P < 0.0001$).

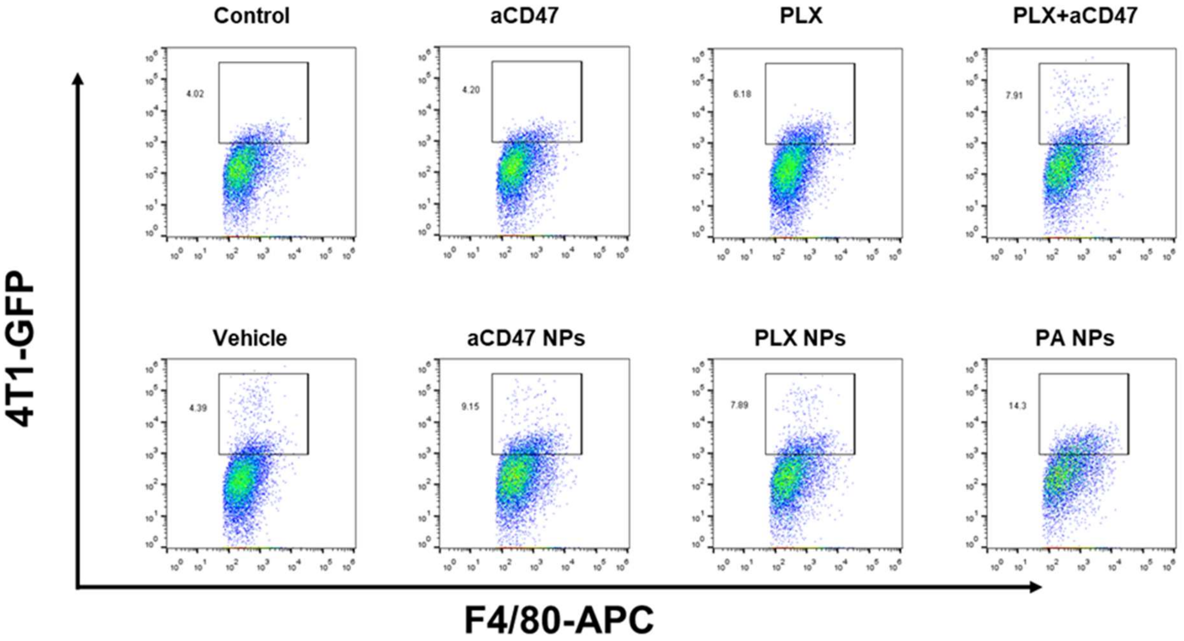

Figure S30. Representative images of flow cytometry analysis of macrophage (F4/80) phagocytosis to 4T1-GFP tumor cells after different treatments.

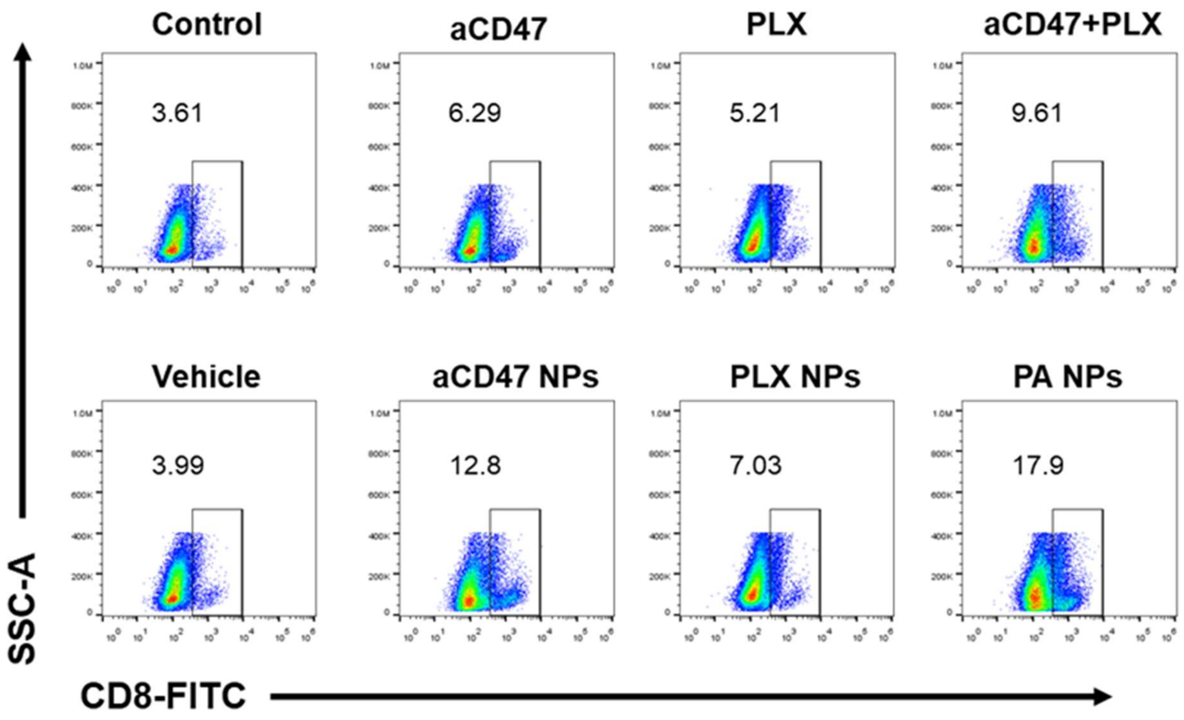

Figure S31. Representative images of flow cytometry of $CD8^+$ T cells (gated on $CD3^+$cells) in tumors after different treatments.

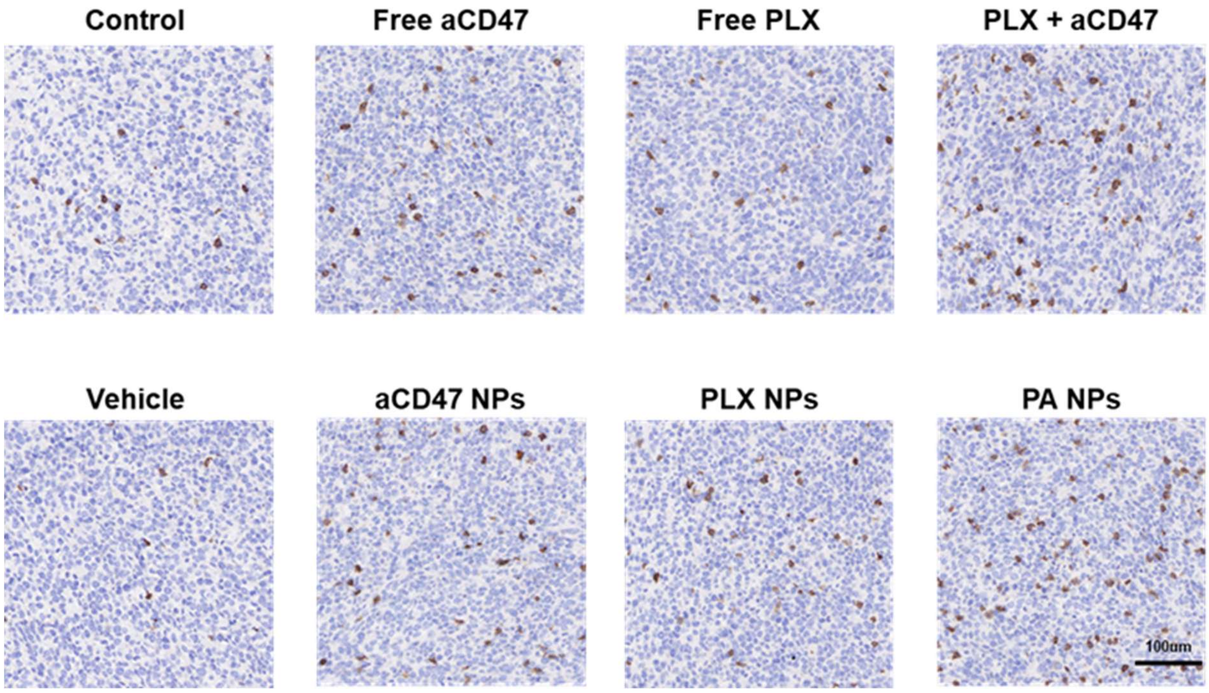

Figure S32. Representative immunohistochemistry images of $CD8^+$ T cell staining of tumor sections after different treatments. The scale bar is 100 μm.

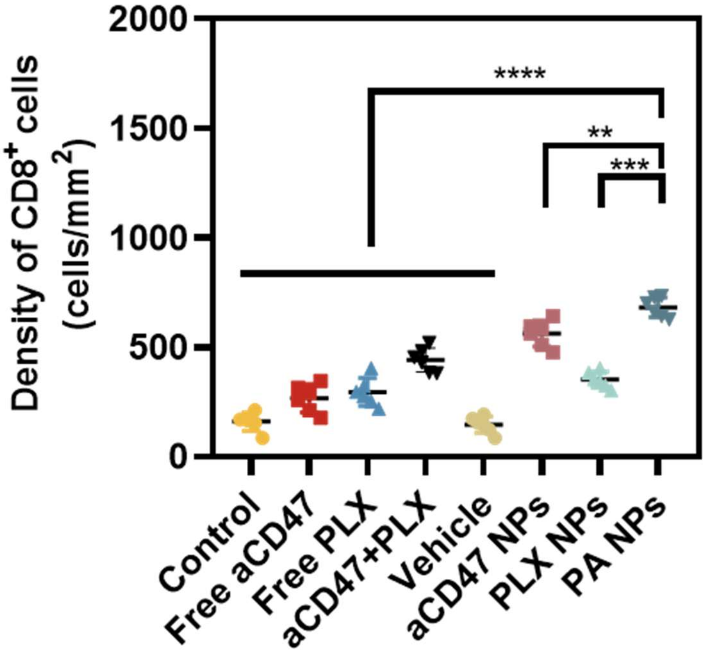

Figure S33. Quantification of $CD8^+$ T cells density in immunohistochemistry images (n = 6). Statistical significance was determined using one-way ANOVA with Tukey's multiple comparison tests. Data are shown as mean ± SD (** $P < 0.01$, *** $P < 0.001$, **** $P < 0.0001$).

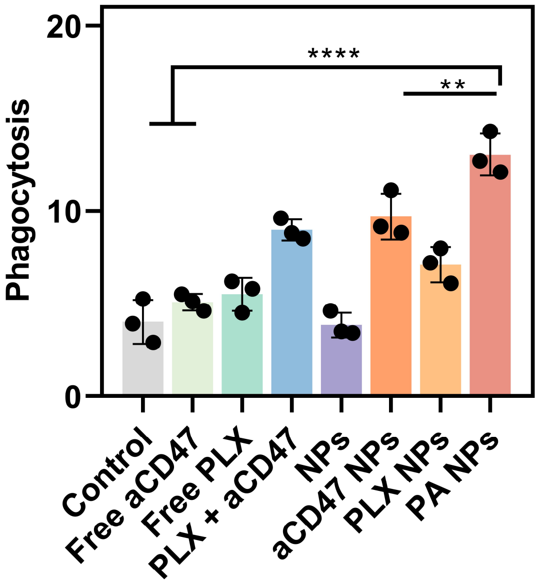

Figure S34. Quantitative analysis of macrophage (F4/80) phagocytosis to EMT6-GFP tumor cells after different treatments (n = 3). Statistical significance was determined using one-way ANOVA with Tukey's multiple comparison tests. Data are shown as mean ± SD (** $P < 0.01$, **** $P < 0.0001$).

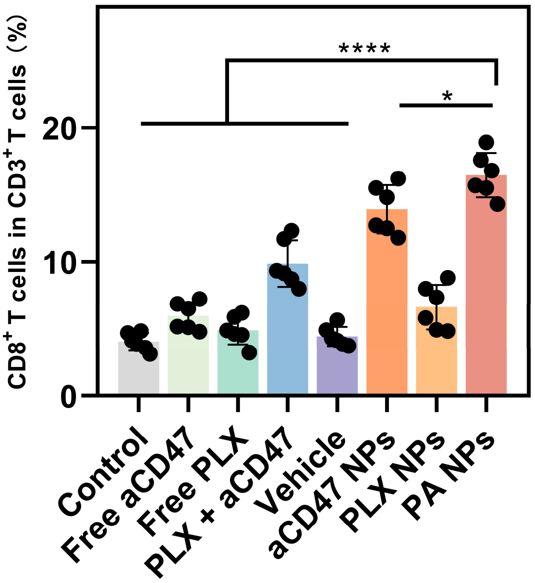

Figure S35. Quantitative analysis of tumor-infiltrating $CD8^+$ in $CD3^+$ T cells in EMT6 tumors after different treatments (n = 6). Statistical significance was determined using one-way ANOVA with Tukey's multiple comparison tests. Data are shown as mean ± SD (* $P < 0.05$, ** $P < 0.01$, *** $P < 0.001$, **** $P < 0.0001$).

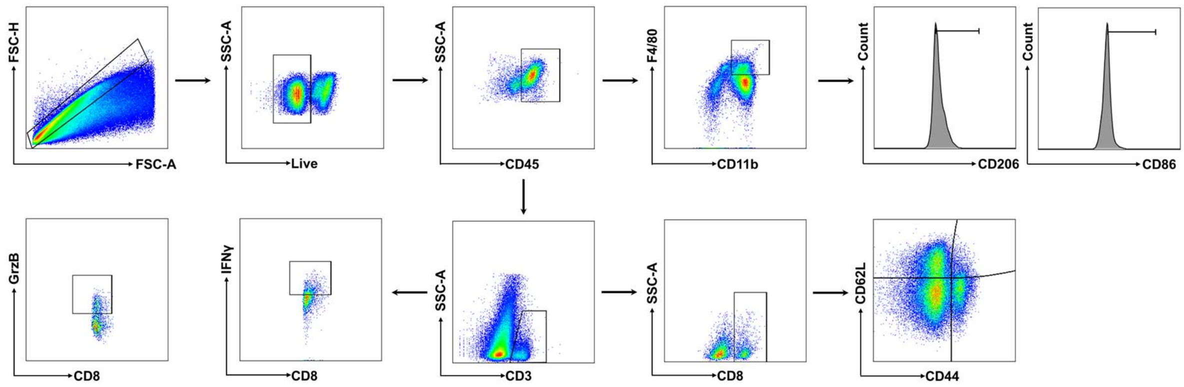

Figure S36. Gating strategies for flow cytometry analysis.

Table S1. PLX3397 and aCD47 encapsulation efficiency and loading capacity of PA NPs (n = 3). Data are presented as means ± SD.

	encapsulation efficiency	loading capacity
PLX3397	91.8 ± 0.2%	5.7 ± 0.01%
aCD47	55.8 ± 1.6%	1.7 ± 0.05%

Table S2. Pharmacokinetic parameters of free IR825 and PA NPs (n = 3, 5mg/kg), equivalent to IR825. Data are presented as means ± SD.

Formulations	$t_{1/2}$ (h)	AUC (mg/ml*h)	clearance rates (ml/h/kg)
IR825	4.37±0.38	0.63±0.11	1.60±0.28
PA NPs	9.68±0.79	2.25±0.23	0.37±0.05

Supplemental references

1. Xu X., Zhang Z., Du J., et al. (2023). Recruiting T-Cells toward the Brain for Enhanced Glioblastoma Immunotherapeutic Efficacy by Co-Delivery of Cytokines and Immune Checkpoint Antibodies with Macrophage-Membrane-Camouflaged Nanovesicles. *Advanced materials (Deerfield Beach, Fla.)* **35**:e2209785. DOI:10.1002/adma.202209785
2. Bai F., Du W., Liu X., et al. (2021). A NO-Responsive Ratiometric Fluorescent Nanoprobe for Monitoring Drug-Induced Liver Injury in the Second Near-Infrared Window. *Analytical chemistry* **93**:15279-15287. DOI:10.1021/acs.analchem.1c02238
3. Zhang Z., Xu X., Du J., et al. (2024). Redox-responsive polymer micelles co-encapsulating immune checkpoint inhibitors and chemotherapeutic agents for glioblastoma therapy. *Nature communications* **15**:1118. DOI:10.1038/s41467-024-44963-3
4. Ma S., Sun B., Duan S., et al. (2023). YTHDF2 orchestrates tumor-associated macrophage reprogramming and controls antitumor immunity through CD8(+) T cells. Nature immunology 24:255-266. DOI:10.1038/s41590-022-01398-6
